# Using gnotobiotic mice to decipher effects of gut microbiome repair in undernourished children on tuft and goblet cell function

**DOI:** 10.1073/pnas.2523178122

**Published:** 2025-11-25

**Authors:** Yi Wang, Hao-Wei Chang, Jiye Cheng, Daniel M. Webber, Hannah M. Lynn, Matthew C. Hibberd, Clara Kao, Ishita Mostafa, Tahmeed Ahmed, Michael J. Barratt, Jeffrey I. Gordon

**Affiliations:** ^a^Edison Family Center for Genome Sciences and Systems Biology, Washington University School of Medicine, St. Louis, MO 63110; ^b^Newman Center for Gut Microbiome and Nutrition Research, Washington University School of Medicine, St. Louis, MO 63110; ^c^Department of Pathology and Immunology, Washington University School of Medicine, St. Louis, MO 63110; ^d^International Centre for Diarrhoeal Disease Research, Dhaka 1212, Bangladesh

**Keywords:** childhood undernutrition, gut microbiome-directed therapeutic foods, barrier function, tuft cells, goblet cells

## Abstract

Studies have implicated perturbations in the postnatal development of the gut microbiome as a contributing factor to childhood undernutrition. Compared to a standard ready-to-use supplementary food, a microbiome-directed complementary food (MDCF-2) designed to repair these perturbations produced superior improvements in ponderal and linear growth in clinical trials of Bangladeshi children with moderate acute malnutrition. Here, “reverse translation” experiments are performed where intact fecal microbiomes collected from trial participants before and at the end of treatment are introduced into female gnotobiotic mice just after delivery of their pups. Pups received diets designed to resemble those consumed by children in the trials to recreate “unrepaired” and “repaired” gut ecosystems. Analyses of the abundances of bacterial strains (metagenome-assembled genomes), their expressed genes, and metabolic products, combined with assessments of ponderal growth and intestinal epithelial lineage transcriptomes (single-nucleus RNA-Seq with follow-up immunocytochemistry) disclosed effects of MDCF-2 associated microbiome repair that cannot be determined, in part because “no treatment” control arms cannot be ethically incorporated into these trials. Specifically, microbiome repair in these mice produced significant increases in ponderal growth, changes in microbial gene expression consistent with a less virulent gut ecosystem and alterations in expression of i) components of cell junctions in the enterocytic and goblet cell lineages, ii) pathways for synthesis and secretion of eicosanoid immune effectors in chemosensory tuft cells, and iii) goblet cell pathways involved in glycosylation and secretion of mucin. Experiments of the type described can help formulate and test hypotheses about how microbiome repair affects host biology.

## Significance

Undernutrition is a global health problem. Recent clinical trials of a gut microbiome-directed complementary food (MDCF-2) designed to repair the perturbed gut microbiomes of undernourished Bangladesh children produced superior growth outcomes versus a standard nutritional supplement. Given ethical considerations and tissue sampling constraints associated with these types of studies, we colonized gnotobiotic mice postnatally with microbiome samples obtained from trial participants before and after treatment to model “unrepaired” and “repaired” gut ecosystems. Using a multiomics approach, we uncover heretofore unappreciated changes in expressed chemosensory tuft cell, mucus-producing goblet cell and absorptive enterocytic functions, and interactions, accompanying microbiome repair. Extending microbiome clinical trials back to preclinical models (“reverse translation”) provides mechanistic insights that can inform design/interpretation of future interventions.

Childhood undernutrition represents a pressing global health challenge. Epidemiologic studies indicate that factors in addition to food insecurity contribute to pathogenesis. One such factor is a disruption in the postnatal assembly and functional maturation of the gut microbiome in undernourished infants/children. This process is largely completed during the first 2 to 3 y after birth in healthy individuals (1). Emerging evidence suggests that proper coordinated codevelopment of the gut microbiome and host organ systems is an important contributor to healthy postnatal growth (2–4).

A randomized controlled clinical trial of 12 to 18-mo-old Bangladeshi children with primary moderate acute malnutrition (MAM) demonstrated that dietary supplementation with a microbiome-directed complementary food (MDCF-2) designed to repair their gut communities significantly improved their ponderal as well as linear growth compared to a commonly used ready-to-use supplementary food (RUSF) formulation, even though the RUSF had 15% higher caloric density than the MDCF (5, 6). DNA and mRNA analyses of fecal samples, serially collected during the course 3-mo long-period of MDCF-2 intervention, disclosed that two *Prevotella copri *(Segatella copri)** strains, whose abundances were positively associated with improvements in participants’ ponderal growth, were the predominant source of transcripts encoding enzymes involved in the metabolism of MDCF polysaccharides (6). A similar result was obtained in a separate randomized controlled clinical trial of 12 to 18-mo-old Bangladeshi children who had presented with severe acute malnutrition (SAM), had undergone a hospital-based initial nutritional resuscitation protocol that was not designed to repair their microbiome and were left with MAM (i.e., they had post-SAM MAM). Compared to RUSF, MCDF-2 treatment in both trials was accompanied by significantly greater changes in the levels of multiple plasma protein biomarkers and mediators of musculoskeletal and CNS development, immune function, and metabolic regulation (7).

Our previous work utilized a gnotobiotic mouse model that involved dam-to-pup transmission of a defined community of age- and growth-associated bacterial strains cultured from Bangladeshi infants/children to dissect the mechanisms underlying MDCF-2’s effects (8). We found that inclusion of *P. copri* in the bacterial consortium significantly increased postnatal weight gain in a MDCF-2-dependent fashion and significantly increased the metabolism of polysaccharides deemed to be key bioactive components of MDCF-2 (8). Moreover, single nucleus RNA-sequencing (snRNA-Seq), combined with targeted mass spectrometric analysis of intestinal segments disclosed that the presence of *P. copri* had a marked effect on metabolism in the predominant gut epithelial cell lineage (enterocytes) (8).

These mechanistic studies used defined culture collections that do not fully capture the complexity of the intact gut luminal ecosystem. In the current report, we compare offspring of gnotobiotic dams colonized with intact uncultured microbiota from children in the upper quartile of clinical response to MDCF-2 in the trial of children with primary MAM. Our goal was to compare the outcomes of MDCF-2 treatment using multiomic analyses to those of a hypothetical scenario in which the undernourished participants had not received any treatment.

## Results

### Experimental Design.

**Fig. 1.**
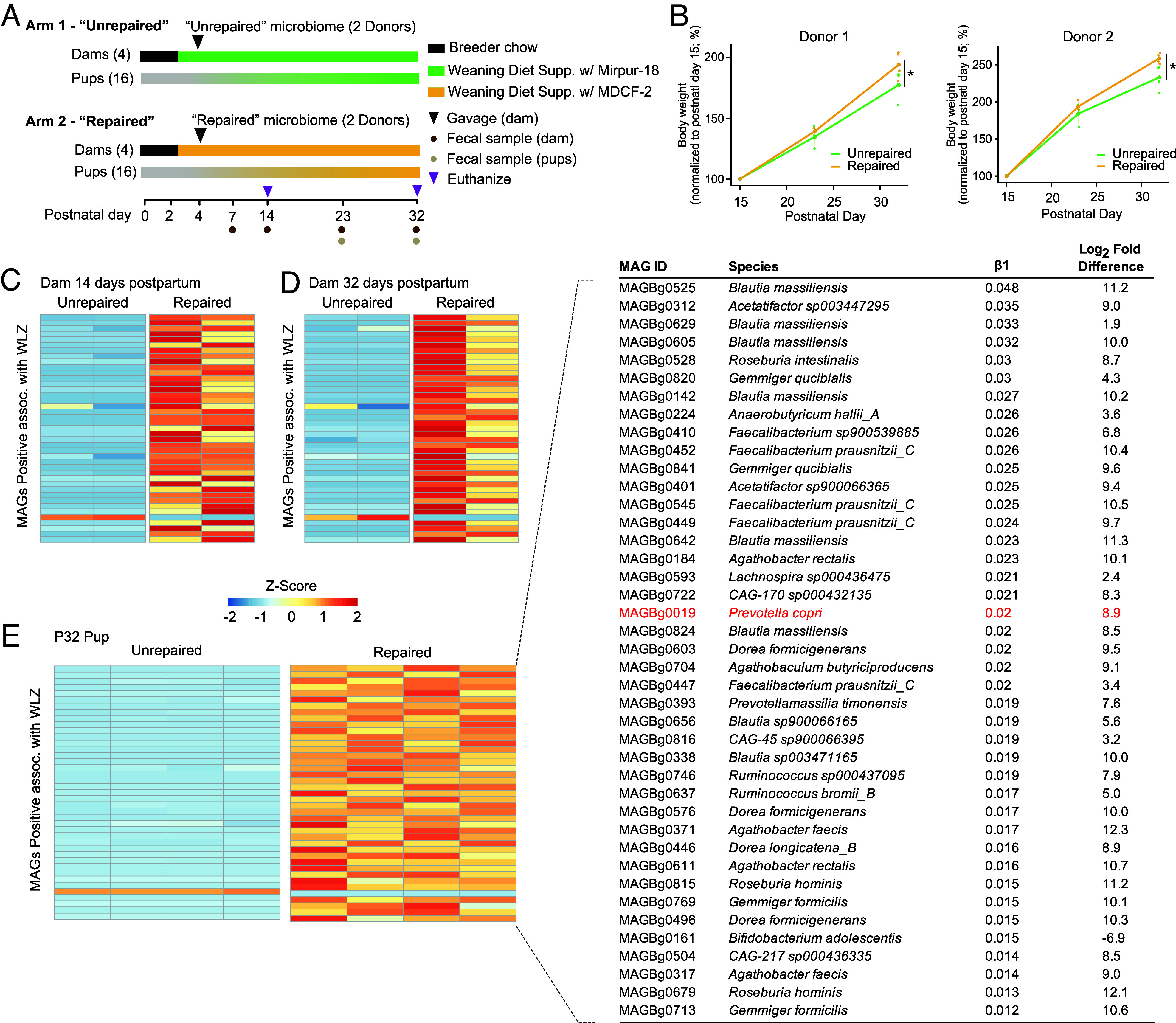
MAG and microbial RNA-seq analyses. (*A*) Experimental design. (*B*) Line plots illustrating relative weight changes from postnatal day 15 (P15) to P32, using P15 as the reference point for comparison. Data are presented for both donor 1 and donor 2 experiments. Error bars represent SD. * indicates statistical significance with *P* < 0.05. (*C*–*E*) Heatmaps representing the relative abundance of colonized bacterial strains (MAGs) positively associated with WLZ in postpartum day 14 (*C*), postpartum day 32 dams (*D*), and postnatal day 32 pups (*E*) for the donor 1 experiment. Z-score was calculated by centering and scaling the log-transformed relative abundances of each MAG across all samples. Each column represents data from an individual animal. The inset in E provides detailed information about the WLZ-associated MAGs shown in the heatmap, including MAG ID, assigned species, β1 coefficient from the linear mixed effects model [WLZ ~ β1(MAG) + β2(study week) + (1|PID)] indicating the strength of WLZ association in the clinical trial, the Log2-fold difference in MAG abundance between “unrepaired” and “repaired” arms of the mouse experiment. The MAGs are ordered by descending β1 coefficient. *Prevotella copri* is highlighted in red.

Fig. 1*A* summarizes the experimental design. Intact uncultured fecal samples that had been stored at −80 °C since their collection were selected from two participants in the randomized controlled clinical study of Bangladeshi children with primary MAM. Among trial participants, these children fell within the upper quartile of ponderal growth response following MDCF-2 treatment, as measured by changes in weight-for-length z-score (WLZ). They also exhibited increases in the abundances of bacterial taxa that were positively associated with WLZ and decreases in taxa negatively associated with WLZ (5, 6). Two fecal samples were used from each child: one collected just before initiating MDCF-2 treatment and one obtained at the conclusion of the 3-month intervention with this therapeutic food. These samples were designated “unrepaired” (pretreatment) and “repaired” (posttreatment) microbial communities. Several custom diets were formulated for these experiments (see Dataset S1 for diet ingredients, nutritional and glycosidic linkage analysis): i) a pre-treatment “weaning diet” reflective of that consumed by 12 to 18-mo-old Bangladeshi children living in the Mirpur urban slum where the randomized controlled clinical trials had been conducted (weaning diet supplemented with Mirpur-18), ii) a “weaning diet” reflecting that consumed by participants in the MDCF-2 arm of the clinical trial (weaning diet supplemented with MDCF-2). Diets were irradiated to ensure their sterility.

Eight germ-free female C57BL/6 J mice that had been maintained on standard breeder chow during their pregnancy delivered a total of 36 pups; nine pups were randomly assigned on postpartum day 1 (P1) to each of four groups of dually housed dams that had been switched to the weaning diet. On postpartum day 2 (P2), two groups of dams received the weaning diet supplemented with Mirpur-18, while the other two groups received the weaning diet supplemented with MDCF-2 (Dataset S1). Two days later (postpartum day 4), each cohoused dam consuming the Mirpur-18-supplemented weaning diet was gavaged with a pretreatment “unrepaired” fecal microbiome sample from one or the other of the two donor children with MAM, while a separate of cohoused dams consuming the weaning diet supplemented with MDCF-2 received the corresponding posttreatment “repaired” community from the same child (total of 4 experimental groups). Thus, maternal-to-pup transfer of these donor communities occurred while the pups experienced a diet sequence that first began with exclusive milk feeding (from the nursing dam) followed by a weaning period where pups consumed the dam’s milk plus the weaning diet supplemented with Mirpur-18 or with MDCF-2 (Dataset S1). Five pups were euthanized in a carbon dioxide chamber (delivered at 30-70% chamber volume displacement per minute), in accordance with the Washington University Animal Euthanasia Policy (https://research.washu.edu/animal-euthanasia-policy/). on postnatal day 14 (P14) from each group. The remaining offspring were maintained with the dams until they were euthanized on P32 (Fig. 1*A*). To control for diet effects, we included two additional germ-free control groups that received either the Mirpur-18–supplemented or the MDCF-2–supplemented weaning diet (n = 2 dams with 11 pups per group).

### Effects on Ponderal Growth.

Mice that received the “repaired” microbiome from either donor demonstrated significantly greater increases in body weight from P15 to P32 than mice that had received “unrepaired” microbiota from the same donor [*P*< 0.05; linear mixed-effects model (mixed effects: group and postnatal day; random effect: mouse); Fig. 1*B*]. As the weaning diet supplemented with MDCF-2 has 10% more energy than the Mipur-18-supplemented diet (Dataset S1), both dietary differences and microbiome configuration could have contributed to the weight gain phenotype. To disentangle these effects, we first assessed the effects of weaning diets supplemented with MDCF-2 versus Mirpur-18 on the ponderal growth of uncolonized (germ-free) mice. Germ-free pups fed the weaning diet supplemented with MDCF-2 exhibited a statistically significant increase in weight gain compared to those receiving the weaning diet supplemented with Mirpur-18 [*P* = 0.014; linear mixed-effects model: relative weight gain ~ diet × days of experiment + (1|mouse)]. We subsequently pooled data from the donor 1- and donor 2-colonized and germ-free mice and applied a linear mixed-effects model [Relative_weight_gain ~ Diet * Day_of_experiment + Microbiome * Day_of_experiment + (1|Mouse_ID)]. This analysis revealed that both the interaction term of microbiome and time of the experiment (*P* = 5.34 × 10^−15^) and the interaction term of diet and time of the experiment (*P* = 0.038) contributed significantly to the observed difference in weight gain. Together, these results indicated that both the MDCF-2-supplemented weaning diet and the repaired microbiome contribute to the observed growth enhancement.

### WLZ-Associated Metagenome-Assembled Genomes (MAGs) in Repaired Versus Unrepaired Microbiomes.

We performed shotgun sequencing of DNAs isolated from i) fecal samples serially collected from dams at postpartum days 7, 14, 23, and 32, ii) fecal samples collected from their pups at P23 and P32 and iii) cecal contents harvested from pups at the time of their euthanasia in a carbon dioxide chamber (delivered at 30-70% chamber volume displacement per minute), in accordance with the Washington University Animal Euthanasia Policy (https://research.washu.edu/animal-euthanasia-policy/) at P32 (Dataset S2). The resulting dataset was used to quantify the representation of 222 MAGs that we had previously found to be significantly positively (n = 75) or negatively (n = 147) associated with ponderal growth (WLZ) in children with primary MAM who participated in the clinical trial (6). Postpartum day 32 dams that received unrepaired or repaired fecal microbiomes from donor 1 contained 107 and 141 WLZ-associated MAGs, respectively (colonization criteria: TPM > 20 and prevalence > 40%). 97.7 ± 5.4% (mean ± SD) and 93.8 ± 0.9% of these MAGs were also present in the cecal microbiomes of their P32 offspring (n = 4 mice/treatment group; Dataset S2*C*). Postpartum day 32 dams colonized with donor 2’s unrepaired and repaired fecal communities contained 111 and 74 WLZ-associated MAGs, respectively, of which 94.4 ± 1.5% and 86.1 ± 7.2% were transmitted to their P32 offspring (n = 4 mice/treatment group; Dataset S2*C*). 63.1% and 92.2% of the transmitted MAGs in donor 1 and donor 2 P32 pup cecal microbiomes were shared across the two experiments (highlighted in Dataset S2*D*). Volcano plots of the WLZ-association coefficient β1 and q-values (*SI Appendix*, Fig. S1*A*) demonstrate that bacterial strains (MAGs) most strongly associated with ponderal growth in the clinical trial had successfully colonized the dams.

This gnotobiotic dam-to-pup transmission model recapitulated the enhanced representation of positive WLZ-associated MAGs in repaired microbiomes; this was evident in dams at postpartum days 14 and 32, and in all of their pups at P32 in both the donor 1 and donor 2 experiments (Fig. 1 *C*–*E* and Dataset S2*C*). In the clinical trial, *P. copri* MAG Bg0019 was a major source of MDCF-2-induced transcripts for carbohydrate-active enzymes (CAZYmes) involved in the metabolism of its bioactive glycan components (6). The abundance of this MAG, which is positively associated with WLZ (6), was significantly greater in the repaired compared to unrepaired microbiomes of dam-pup dyads that had received either the donor 1 or the donor 2 community (Fig. 1*E*).

### Transcriptional Responses of Small Intestinal Epithelial Cell Lineages to Microbiome Repair.

**Fig. 2.**
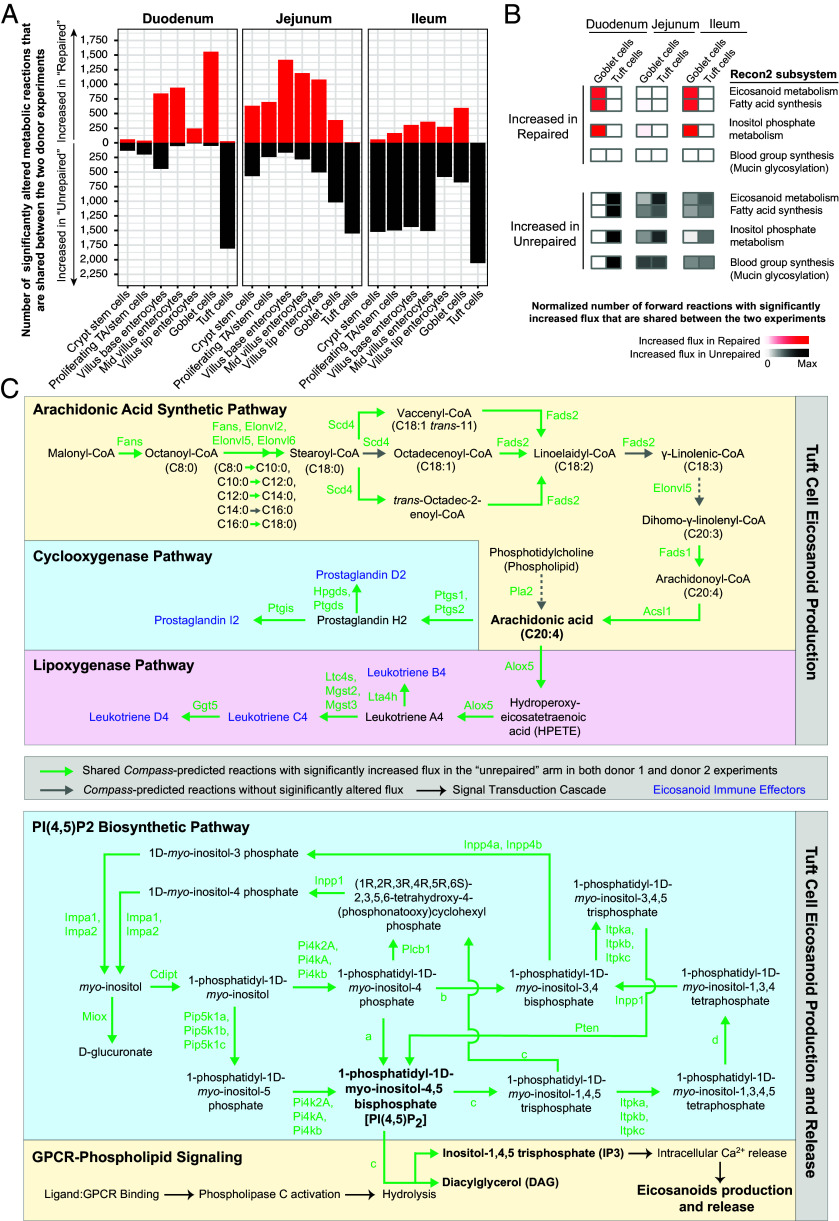
Compass-based analysis of tuft cell metabolism in mice modeling unrepaired and repaired microbiomes. (*A*) Bar plot showing the number of metabolic reactions with significantly altered flux, as predicted by *Compass*, shared between donor 1 and donor 2 experiments. Data are presented for each segment of small intestine. (*B*) Heatmap representing the normalized number of Compass-predicted forward metabolic reactions with significantly altered flux that are shared between donor 1 and donor 2 experiments. Data are presented for select Recon2 metabolic subsystems in tuft cells and goblet cells from the duodenum, jejunum, and ileum. The blood group synthesis subsystem includes glycosylation reactions that are also involved in mucin glycosylation, as indicated by the parenthesis. Red indicates the normalized number of reactions with increased flux in the repaired arm while black indicates the normalized number of reactions with increased flux in the unrepaired arm. Color intensity represents the number of reactions with significantly increased flux in a given Recon2 subsystem within a specific cell type of an experimental arm, normalized to the total number of such reactions across all epithelial cell clusters in the same arm. The term “max” in the color code refers to a normalized value of 1. (*C*) *Top* panel is a schematic of tuft cell metabolic pathways predicted by Compass to exhibit significantly altered flux in response to unrepaired and repaired microbiomes. These pathways include arachidonic acid synthesis, prostaglandin synthesis (via the cyclooxygenase pathway), and leukotriene synthesis (via the lipoxygenase pathway). *Bottom* panel is a schematic representation of metabolic pathways involved in PIP2 synthesis and GPCR-phospholipid signaling in tuft cells, which facilitate the release of eicosanoid immune effectors. Enzyme group ‘a’ includes Pikfyve, Pip4k2a, Pip4K2b, Pip4K2c, Pip5k1a, Pip5k1b, Pip5k1c. Enzyme group ‘b’ includes Hcst, Pik3c2a, Pik3c2b, Pik3c2g, Pik3ca, Pik3cb, Pik3cd, Pik3cg, Pik3r1, Pik3r2, Pik3r3, Pik3r5. Enzyme group ‘c’ includes Plcb1, Plcb2, Plcb3, Plcb4, Plcd1, Plcd3, Plcd4, Plce1, Plcg1, Plcg2, Plch1, Plch2, Plcl1, Plcxd2, Plcz1. Enzyme group ‘d’ includes Inpp5a, Inpp5b, Inpp5d, Inpp5e, Inpp5j, Inppl1, Synj1. Green arrows indicate shared reactions with significantly increased flux in duodenal, jejunal, and ileal tuft cells in mice belonging to the “unrepaired” arm in both donor 1 and donor 2 experiments. Gray arrows represent reactions without significant predicted flux changes. Black arrows denote the signal transduction cascade associated with GPCR-phospholipid signaling. Blue text highlights the eicosanoid immune effectors synthesized in tuft cells. Green text represents enzymes involved in catalyzing the metabolic reactions.

To assess the intestinal epithelial response to microbiome repair along the crypt-to-villus and duodenal-to-ileal axes of the intestine, single-nucleus RNA sequencing (snRNA-Seq) was performed on duodenal, jejunal, and ileal segments harvested from 3 animals in each of the four treatment groups (Dataset S3). Enterocytes, enteroendocrine cells, goblet cells, tuft cells, Paneth cells, transient amplifying and stem cells, plus intraepithelial lymphocytes were identified based on expression of known marker genes (*SI Appendix*, Fig. S1 *B* and *C*). Enterocytes were further classified into three subclusters corresponding to their location at the base, middle, and tips of villi (*SI Appendix*, Fig. S1*C*). Pseudobulk analysis (*Methods*) was used to identify genes that were significantly differentially expressed in mice receiving unrepaired compared to repaired fecal microbiomes from donors 1 and 2 [threshold cutoffs: FDR-adjusted *P*-value (q-value) < 0.1; fold-difference in transcript levels > 1.5] (Dataset S3*B*). We then used the *Compass* algorithm (9) to assess the effects of MDCF-2-mediated microbiome repair on the differential expression of metabolic pathways in seven epithelial cell clusters identified in our snRNA-Seq datasets. Compass employs in silico flux balance analysis to predict metabolic reaction rates based on mass balance constraints and enzymatic capacity for each metabolic reaction curated in the Recon 2 reconstructed model of human metabolism (10). We focused on reactions inferred to have significantly altered flux with consistent directionality of change across both donor 1 and donor 2 experiments (Fig. 2*A*).

Across all three regions of the small intestine, multiple epithelial cell types exhibited high degree of overlap in their Compass-predicted metabolic changes across the two donor experiments (Dataset S3*C*). In mice that received repaired microbiomes, crypt stem cells, proliferating TA/stem cells, and the three enterocyte clusters had transcriptional changes indicative of increased activities of pathways related to glutamate metabolism and fatty acid oxidation— two key sources of energy for these cells (11–13) (*SI Appendix*, Fig. S2 and Dataset S3*D*).

#### Tuft cell response.

Tuft cells play a key role in modulating mucosal immunity, in part by secreting eicosanoid immune effectors including leukotrienes and prostaglandin D_2_ (14). *Compass* analysis revealed that tuft cells from mice colonized with unrepaired microbiomes exhibited increased flux through multiple metabolic pathways involved in the biosynthesis and release of these effectors; they include elevated activities in the fatty acid metabolism, eicosanoid metabolism, and inositol phosphate metabolism subsystems (Fig. 2*B* and *SI Appendix*, Fig. S2). Specifically, flux in reactions leading to the synthesis of arachidonic acid, a common precursor for synthesis of leukotrienes and prostaglandins, was increased (Fig. 2*C*). Downstream of arachidonic acid, reactions leading to the synthesis of prostaglandins and leukotrienes were predicted to have significantly increased flux (Fig. 2*C*). Release of these eicosanoid effectors depends on an upstream GPCR signaling cascade that involves a critical metabolic reaction in which 1-phosphatidyl-1D-*myo*-inositol-4,5-bisphosphate [PI(4,5)P_2_] is hydrolyzed to second-messengers inositol-1,4,5 triphosphate (IP_3_) and diacylglycerol (DAG) (15). IP_3_ binds to intracellular calcium channels in the endoplasmic reticulum, causing release of Ca^2+^ into the cytoplasm, which in turn triggers release of eicosanoid immune effectors. The snRNA-Seq data-derived *Compass*-based analysis of tuft cells indicated enhanced flux toward biosynthesis of PI(4,5)P_2_, the precursor for IP_3_, in the unrepaired donor 1 and donor 2 arms across all three small intestinal segments (Fig. 2*C*).

We used targeted ultrahigh performance liquid chromatography-triple quadrupole mass spectrometry (*Method*s) to assay prostaglandins (D_2_, E_2_, I_2_) and leukotrienes (B_4_, C_4_, D_4_) in duodenal, jejunal, and ileal segments (n = 4 mice/arm; 4 arms). However, all these compounds were below the limit of detection (<45 ng/mg tissue), likely reflecting the fact that tuft cells are sparsely represented (0.4 to 2% of cells) in the small intestinal epithelium (16).

**Fig. 3.**
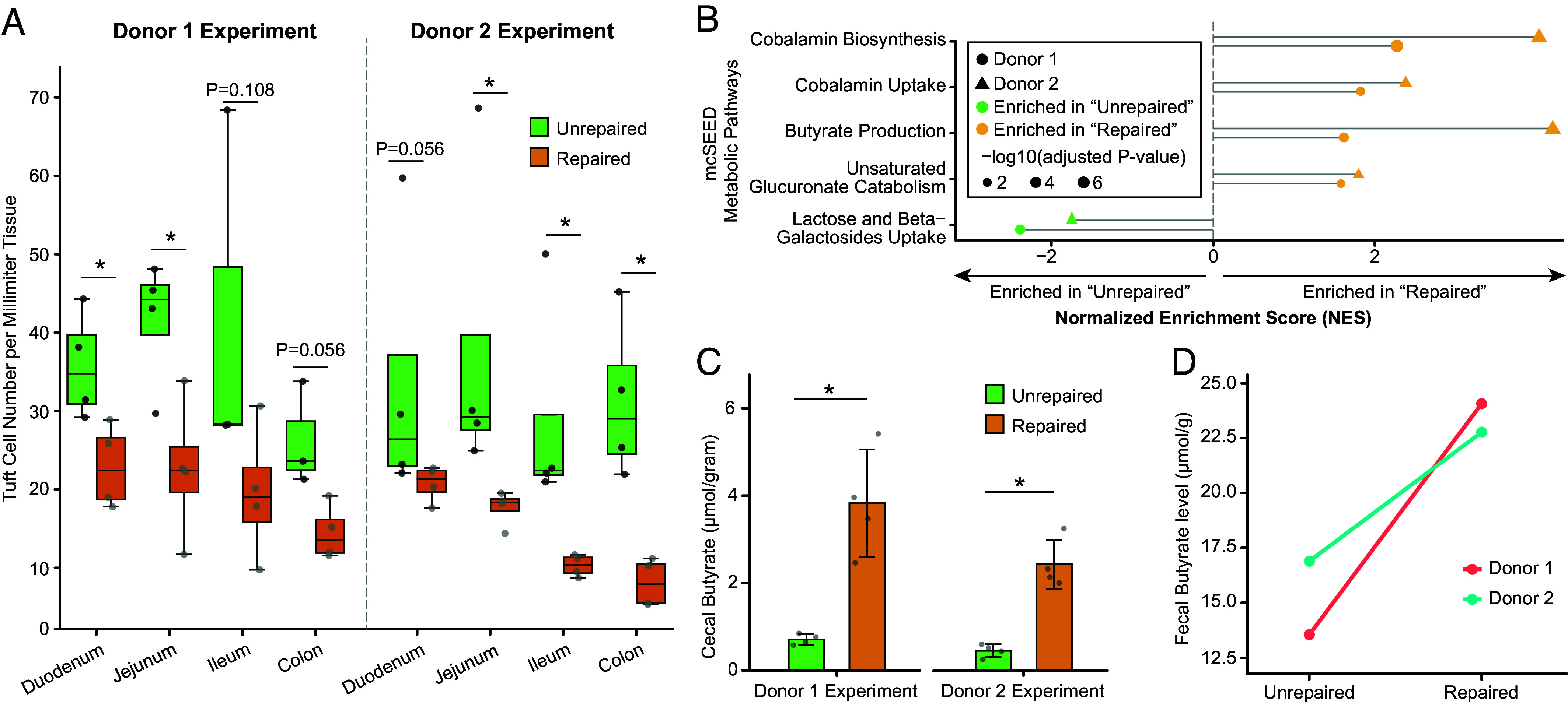
Tuft cell hyperplasia and cecal butyrate level changes in response to an “unrepaired” gut ecosystem. (*A*) Quantification of tuft cell density across different intestinal regions based on whole-slide scanned images of DCLK1-immunostained sections. Data are presented as box and whisker plots for each donor and intestinal segment. *P < 0.05 (Mann–Whitney *U* test). (*B*) Lollipop plot illustrating the normalized enrichment score (NES) for mcSEED metabolic pathways with significantly altered expression from microbial RNA-seq data, as identified from GSEA. The length of each lollipop represents the NES, the color indicates the arm (unrepaired” or repaired) in which the pathway is enriched, and the size of the dots reflects the level of statistical significance of the enrichment (adjusted *P*-value). (*C*) Cecal butyrate levels (μmol/g cecal contents). (*D*) Fecal butyrate levels (μmol/g feces) in samples obtained from donor 1 and donor 2 prior to MDCF-2 treatment (unrepaired) and at the conclusion of the 3-mo intervention (repaired).

Activation of G protein–coupled receptor (GPCR) signaling in tuft cells can form a positive feedback loop to promote tuft cell differentiation and hyperplasia from intestinal stem cells (17–19). Using antibodies to Doublecortin-Like Kinase 1 (Dclk1), we quantified the number of tuft cells in sections prepared from the duodenum, jejunum, ileum and colon (n = 4 mice/treatment group; 4 treatment groups). The number of tuft cells was significantly increased in the duodenum and jejunum of mice colonized with the unrepaired compared to repaired microbiome from donor 1 (Mann–Whitney U Test; *P* = 0.015 and 0.030, respectively), with a trend toward increased numbers in the colon (*P* = 0.056) (Fig. 3*A* and Dataset S4). Tuft cell number was significantly increased in the jejunum (*P* = 0.015), ileum (*P* = 0.015), and colon (*P* = 0.015) in mice harboring the unrepaired microbiome from donor 2, with a trend toward an increase in the duodenum (*P* = 0.056) (Fig. 3*A* and Dataset S4).

#### Expression of metabolic pathways in the transplanted microbiomes.

We performed an in silico analysis to define the representation of 158 metabolic pathways described in microbial community SEED (mcSEED) in all MAGs (Dataset S5) (20). We used the findings to interpret the results of microbial RNA sequencing of cecal contents collected from pups on P32 (n = 4 animals per arm; 2 arms per donor experiment; Datasets S5 *A* and *B*). Gene Set Enrichment Analysis (GSEA) was then applied to identify mcSEED pathways whose expression was significantly enriched (*q*-value < 0.1) in the MAG-derived metatranscriptomes. We focused on pathways that were significantly altered by microbiome repair in both transplanted donor 1 and donor 2 communities (Dataset S5*C*).

Transcripts assigned to the “lactose and beta-galactosides uptake” pathway were enriched in the metatranscriptomes of the transplanted *unrepaired* donor microbiomes (Dataset S5*C*). Consistent with their known role in lactose utilization, MAGs assigned to *Bifidobacterium longum* and *Bifidobacterium breve* were primary contributors of this pathway, accounting for 10% and 20% of the leading-edge transcripts in the donor 1 experiment, and 12.5% and 37.5% in the donor 2 experiment, respectively (Dataset S5*D*). These *Bifidobacterium* species are typically early colonizers of the infant gut, with their representation normally diminishing during the transition from exclusive milk feeding to a weaning diet (21–24). All MAGs assigned to *B. longum* and *B. breve* showed significant *negative* correlations with WLZ in the clinical study performed in 12 to 18-mo-old children with MAM (Dataset S5*D*).

In contrast, both repaired microbiomes were enriched for transcripts involved in i) butyrate production, ii) unsaturated glucuronate catabolism and iii) cobalamin biosynthesis and uptake (Fig. 3*B* and Dataset S5*C*). Butyrate serves as an important energy source for enterocytes (25) and has anti-inflammatory effects including suppression of NF-κB signaling (26), restriction of tuft cell differentiation and type II immunity (27) and enhancement of gut barrier function (28). The increased “unsaturated glucuronate metabolism” in the repaired microbiome could reflect enhanced bacterial utilization of glucosaminoglycan-derived uronates, a process described for gut bacteria that metabolize Δ4,5-unsaturated uronic acids (including Δ4,5-unsaturated glucuronic acid or Δ4,5-unsaturated iduronic acid) liberated from mucosal or dietary glycans (29). Cobalamin and its component corrinoids have important roles in bacterial metabolism and communication, impacting microbial–microbial and microbial–host interactions (30). MAGs assigned to species that contributed to each of these enriched pathway’s leading-edge transcripts are described in Dataset S5*D* which also includes information about whether the MAGs were significantly positively or negatively associated with WLZ in the clinical trials.

Butyrate is a key suppressor of tuft cell activation (27). Consistent with this finding, cecal butyrate concentrations, quantified by gas chromatography-mass spectrometry, were significantly elevated in the repaired compared to unrepaired community contexts for both donors (Fig. 3*C*), while other short-chain fatty acids and succinate were not affected (Dataset S6). Fecal butyrate levels in both donors were also increased at the end of MDCF-2 treatment (Fig. 3*D*). Our previous report (6) showed that the mcSEED pathway for butyrate production was significantly enriched in MAGs with high WLZ-association coefficients, further underscoring the clinical relevance of this finding.

#### Goblet cell response.

**Fig. 4.**
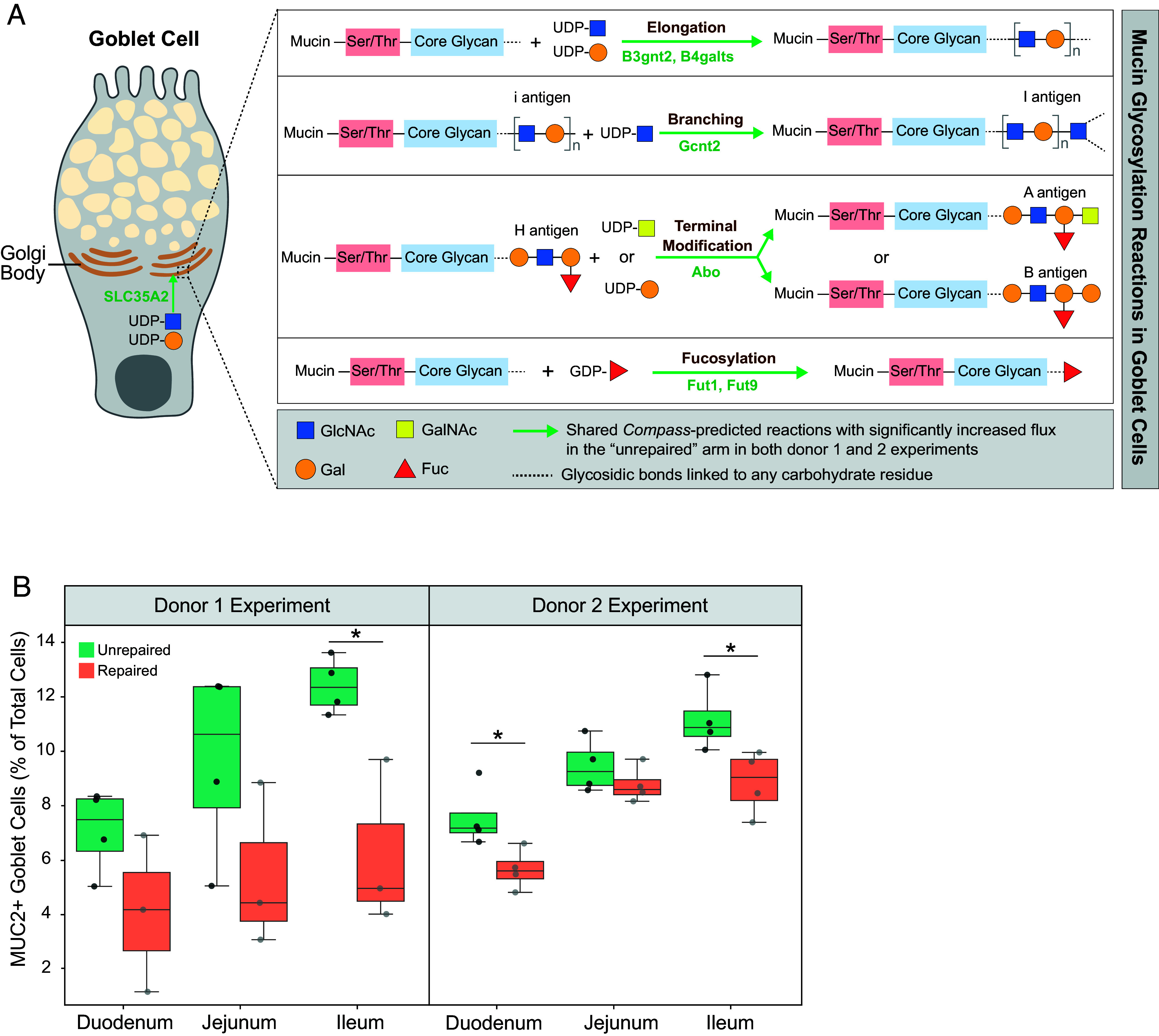
Goblet cell hyperplasia and mucin glycosylation in response to “unrepaired” gut ecosystem. (*A*) Schematic representation of four primary types of mucin glycosylation reactions predicted by *Compass* to have significantly increased flux in goblet cells exposed to the “unrepaired” microbiome. These include glycan elongation, branching, terminal modification, and fucosylation. Abbreviations: GlcNAc, N-acetylglucosamine; GalNAc, N-acetylgalactosamine; Gal, Galactose; Fuc, Fucose; Ser/Thr, Serine/Threonine. Green arrows indicate shared reactions with significantly increased flux in jejunal and ileal goblet cells present in the “unrepaired” arm across both donor 1 and donor 2 experiments. Gray arrows represent reactions with no significant changes in flux. Green text represents enzymes involved in catalyzing the metabolic reactions. (*B*) Goblet cell density across three intestinal regions based on whole slide scanned images of MUC2 immunostaining. Data are presented as box and whisker plots for each donor and intestinal segment. *P < 0.05 (Mann–Whitney *U* test).

Prostaglandin D_2_ produced by tuft cells is known to act on goblet cells to boost their mucus production and secretion (31, 32). Immunostaining of MUC2 disclosed a significant increase in goblet cell density in mice colonized with unrepaired microbiomes from donors 1 and 2 (Fig. 4 and Dataset S7; n = 1 section per intestinal region, 4 mice per arm; 2 arms per donor experiment).

Mucin, the principal component of the mucus layer, undergoes extensive, multistep glycosylation in the Golgi apparatus of goblet cells before secretion; these steps are critically linked to its barrier-protecting properties (33, 34). Compared to mice colonized with repaired microbiomes, jejunal and ileal goblet cells from mice harboring unrepaired communities exhibited significantly greater enzymatic flux in the Recon 2 subsystem annotated as “blood group biosynthesis”; this subsystem encompasses the same glycosylation reactions used in the production of mucin (Fig. 2*B*). The first reaction involves enhanced mucin glycan chain elongation mediated by β-1,4-galactosyltransferases (B4GALT) and β-1,3-N-acetylglucosaminyltransferase 2 (B3GNT2). B4GALT incorporates galactose (Gal) residues, while B3GNT2 adds N-acetylglucosamine (GlcNAc) residues, together forming repeating disaccharide units [-Galβ(1,4)-GlcNAcβ(1,3)-] (34). *Compass* also predicted enhanced transport of the two essential nucleotide-sugar donors, UDP-Gal and UDP-GlcNAc into goblet cell Golgi compartments via the transporter SLC35A2 (Fig. 4*A*). The second type of reaction identified by *Compass* as increased in the goblet cells of mice colonized with the unrepaired microbiomes involves N-acetylglucosaminyltransferases (GCNTs), which facilitate glycan branching by adding N-acetylglucosamine (GlcNAc) residues to existing i-antigen structures in mucin glycans (Fig. 4*A*). The third category of glycosylation reactions predicted to be increased in mice that modeled the unrepaired microbiome state is related to the formation of A and B antigen structures as a means of terminal modifications on mucin glycan chains. Catalyzed by A and B transferases encoded by the Abo gene, N-acetylgalactosamine (GalNAc) and galactose (Gal) residues are transferred to the H antigen, respectively. The presence of these ABH antigens in glycan termini influences mucosal interactions with microbes (33). The fourth type of reaction involves fucosylation, catalyzed by members of the fucosyltransferase (FUT) family. Despite being a minor component of mucin glycans, fucose is vital for enhancing mucus viscoelasticity and forming key terminal mucin glycan structures (33).

Goblet cells rely on GPCR-phospholipid signaling to trigger mucus secretion. In mice that received both unrepaired microbiomes, jejunal goblet cells exhibited predicted increased metabolic flux toward the production of IP3 and DAG (*SI Appendix*, Fig. S3). IP3 stimulates the release of Ca^2+^ from the endoplasmic reticulum, a signaling event that is crucial for mucus secretion from goblet cells (15, 35).

Expansion of tuft and goblet cells in the gut epithelium is commonly associated with host defense mechanisms triggered by enteric parasites (17–19). To exclude parasite infection as a contributing factor, we aligned shotgun sequencing reads from cecal contents harvested from P32 mice to EuPathDB (release 48) (36), a curated genomic database containing 388 eukaryotic pathogens that includes intestinal parasites such as protists that are capable of inducing tuft cell responses (17–19). Metagenomic reads were prefiltered to remove host and bacterial MAG sequences prior to alignment. None of the samples contained detectable levels of the eukaryotic pathogens represented in this database (*Methods;* n = 4 animals treatment arm; 4 arms in total; 16 samples). As noted above, microbial RNA-seq analysis revealed significant upregulation of butyrate production pathways in bacterial MAGs from the repaired microbiomes (Fig. 3*B* and Dataset S5*C*). Together, these findings support the notion that shifts in bacterial metabolism, rather than parasitic infection, underlie the tuft cell response observed in our gnotobiotic model.

### Effects on Expression of Virulence Factors and Components of Epithelial Junctions.

**Fig. 5.**
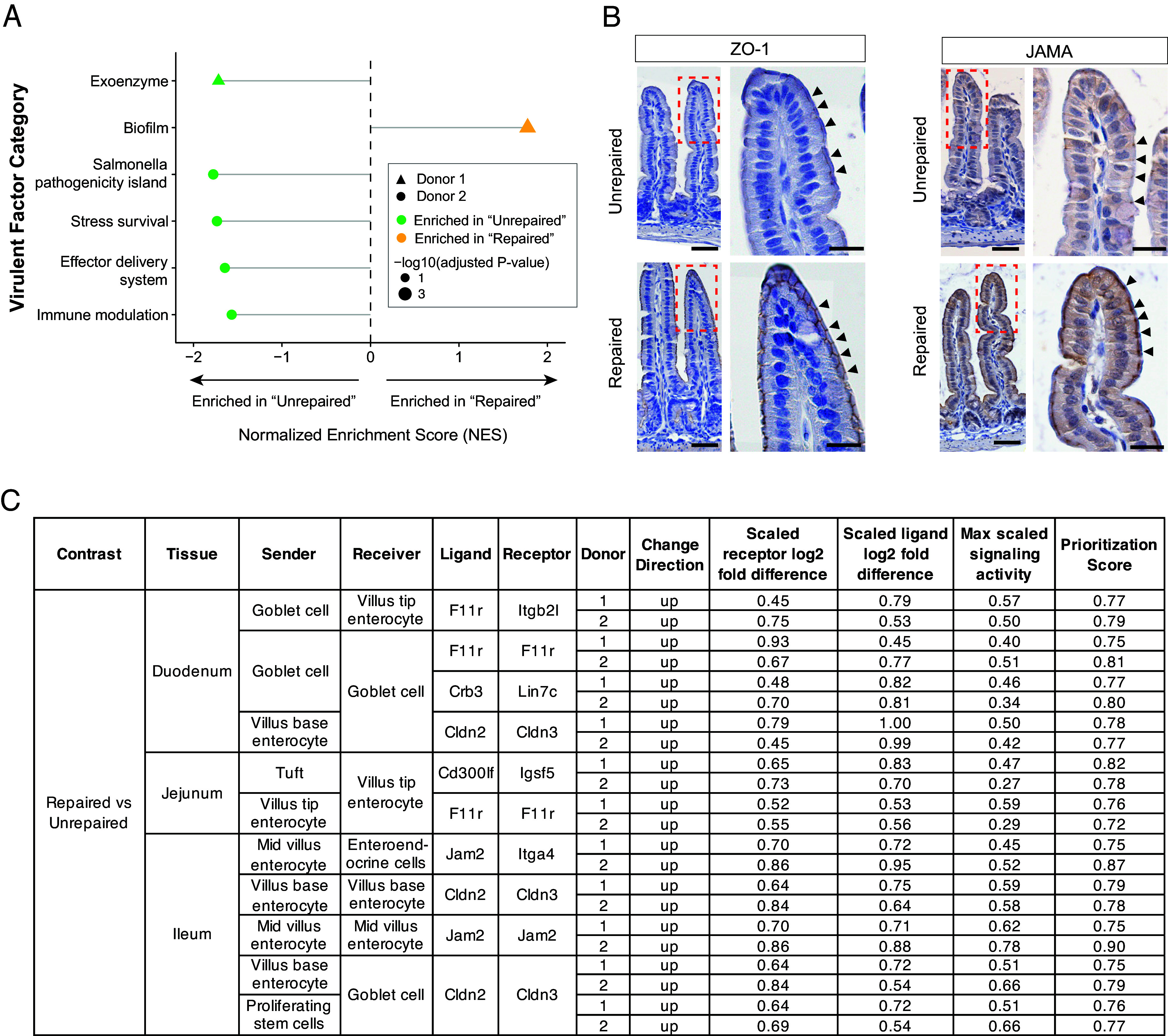
Alterations in microbial virulence and epithelial cell tight junctions. (*A*) Lollipop plot of NES for virulence factor categories from VFDB with significant altered expression, as identified by GSEA from microbial RNA-seq data. (*B*) Representative immunostaining images showing the distribution of tight junction components ZO-1 and JAMA in the jejunums of P32 mice. Brown staining indicates positive immunoreactive signal, while blue staining is from nuclei counterstained with hematoxylin. (Scale bar, 50 μm in the *Left* panels and 20 μm the *Right* panels.) (*C*) Table summarizing the shared epithelial tight junctional interactions that ranked in the top 10% prioritization score across the duodenum, jejunum, and ileum in mice from both donor 1 and donor 2 experiments.

We used DIAMOND (37) to align the protein sequences of virulence factors from the Virulence Factor Database (VFDB) to the protein-coding sequences of MAGs in cecal and fecal samples from postpartum day 32 dams and their P32 pups that satisfied colonization criteria based on abundance and prevalence (*Methods*). A MAG was deemed “putatively virulent” if it encoded both an exotoxin and an effector delivery system component (DIAMOND alignment criteria: bitscore >200 and E-value < 10^−100^; Dataset S2*E*). The results disclosed that samples collected from animals with either of the two donors’ unrepaired microbiomes contained a significantly greater number of MAGs satisfying these virulence factor criteria than the corresponding repaired microbiomes (*SI Appendix*, Fig. S4*A*). GSEA of the microbial RNA-Seq datasets revealed significantly enriched expression of VFDB pathways involved in i) host extracellular matrix degradative functions, ii) effector delivery systems (injection of virulence factors into host cells), iii) immune modulation (host immune system evasion, iv) stress survival and v) a *Salmonella* pathogenicity island in cecal samples obtained from mice harboring the unrepaired microbiomes (Fig. 5*A* and Dataset S5 *E* and *F*). Biofilm formation was the only VFDB pathway enriched in a repaired microbiome (Fig. 5*A* and Dataset S5 *E* and *F*). Although biofilms support bacterial persistence, they are not necessarily indicative of a state of increased virulence.

Immunostaining for epithelial tight junction biomarkers ZO-1 and JAMA (encoded by the F11r gene) showed decreased levels of both proteins in the duodenums, jejunums, and ileums of mice colonized with the two unrepaired donor microbiomes (Fig. 5*B*). In contrast, levels of both proteins were comparable in jejunal tissues from P32 germ-free mice fed weaning diets supplemented with either Mirpur-18 or MDCF-2, indicating that these tight junctional changes were not driven by diet alone (*SI Appendix*, Fig. S4*B*).

We then applied the “MultiNicheNet” algorithm (38) to the snRNA-Seq dataset to further examine the correlation between microbiome repair and expression of tight junction components that could mediate interactions between various epithelial cell types along the crypt-villus and duodenal to ileal axes of the small intestine (Dataset S3*E*). “MultiNicheNet” infers differentially active ligand–receptor interactions between experimental conditions. For every ligand–receptor pair within a sender–receiver cell type combination, a prioritization score is computed based on parameters described in *Methods*. The higher the prioritization score, the more confidently the model predicts that this ligand–receptor pair is altered between experimental arms (38). For our analysis, we tested all possible epithelial cell-to-cell interactions by designating every epithelial cell type as both a sender and a receiver; we focused on ligand–receptor interactions that ranked in the top 10% prioritization score in both donor 1 and donor 2 experiments. The results are summarized in Fig. 5*C* and indicate that repair is accompanied by increased expression of junctional components, including JAMs and Claudins, operating between enterocytes themselves, between enterocytes and tuft cells, goblet cells, and enteroendocrine cells, as well as between goblet cells themselves and goblet and transit amplifying cells. Remarkably, the effects of microbiome repair on expression of junctional components varies as a function of cellular position along the crypt-to-villus axis (e.g. Cldn2, expressed in enterocytes located at the base of villi) as well as the duodenal–ileal axis (JAMA, restricted to the duodenum and jejunum). Collectively, these results provide evidence supporting a relationship between microbiome repair and fortification of the gut epithelial barrier.

## Discussion

We have previously described the development of a microbiome-directed complementary food (MDCF-2) that was superior to a standard ready-to-use supplementary food at restoring healthy ponderal and linear growth in randomized controlled clinical trials of undernourished Bangladeshi children (5, 7, 39). MDCF-2 was designed to include plant ingredients that are enriched in specific glycan structures that are metabolized by growth-associated gut bacteria taxa, including specific strains of *P. copri* (4, 6, 8). In the present study, we employed a “reverse translation” approach that used gnotobiotic mice colonized with intact uncultured fecal samples from two Bangladeshi children with MAM enrolled in one of these trials. The experimental design featured an “unrepaired arm” where a group of gnotobiotic pups received, from their dams, a fecal microbiome sample obtained from a trial participant just before initiation of MDCF-2 treatment, and a “repaired arm” where a corresponding group of pups received a microbiome sample obtained from the same child on the last day of treatment. The cellular and molecular effects of the unrepaired and repaired communities from the two children with MAM on gut biology was examined in the context of diets representative of those consumed by the children at the time of their microbiome sampling. The two human donors used in this preclinical study were selected based on their robust ponderal growth responses (upper quartile of trial participants receiving MDCF-2), and the accompanying robust change in the representation of growth-associated bacterial taxa.

Compared to mice receiving repaired microbiomes, animals that received the unrepaired pretreatment communities exhibited features of a more virulent gut luminal ecosystem that were reflected in the responses of intestinal tuft cells and goblet cells. These features included i) increased expression of virulence factors by bacterial members of the microbiome, ii) reduced production of butyrate, a key suppressor of tuft cell activation by the microbiome, iii) increased number of tuft cells and accompanying increases in their expression of genes involved in synthesis and metabolism of eicosanoid immune effectors, plus iv) increased expression of multiple steps in mucin glycosylation in goblet cells. Tuft cells are known to play an important role in type 2 immunity, particularly in response to parasitic infections (17–19). Upon sensing parasites, they secrete IL-25 (17–19), leukotrienes (40–42), and prostaglandin D_2_ (43), which in turn activate group 2 innate lymphoid cells (ILC2s). Activation leads to secretion of type 2 cytokines IL-4/13, which act on goblet cells to promote goblet cell hyperplasia (17) and glycosylation of mucin (44). In addition, Prostaglandin D_2_ produced by activated tuft cells directly enhances goblet cell mucus production (31, 32). Chronic activation of tuft cell–mediated immunity, as observed in parasitic infection models, has been linked to moderate weight loss (14), suggesting a potential mechanism underlying the reduced weight phenotype observed in our experiments.

To date, only a limited number of metabolites, including butyrate, succinate, and N-decanoylglycine, have been shown to modulate tuft cell number/biology. Our study documented an increase in levels of butyrate, a known inhibitor of tuft cell expansion, under microbiome repaired compared to unrepaired conditions. There were no significant differences in levels of succinate, a well-characterized metabolite that activates tuft cells. N-decanoylglycine was recently identified as a tuft cell–expanding metabolite in a *Shigella* infection model (32); it was below the limits of detection by LC-QqQ-MS in mice belonging to all arms in our experiments. A small number of studies have begun to implicate tuft cell biology in the context of malnutrition, but significant gaps remain. For example, tuft cell numbers were markedly increased in marmosets with Marmoset Wasting Syndrome (45). Moreover, in gnotobiotic mice colonized with a consortium of bacterial strains cultured from the fecal microbiota of a stunted Malawian infant and fed a representative Malawian infant diet supplemented with sialylated milk oligosaccharides there were i) increases in intestinal succinate levels and in the number of tuft cells, ii) activation of a succinate-induced pathway in tuft cells linked to Th2 responses and iii) reductions in osteoclastogenic activity and bone resorption. These effects were not observed in germ-free animals fed the same supplemented diet, highlighting the critical role played by the microbiome in mediating this tuft cell phenotype (46).

We chose to use a gnotobiotic model that involved maternal-to-pup microbiome transmission rather than one that employed direct introduction of microbial communities into already weaned animals. We did so because early-life colonization is a critical window for intestinal and immune maturation. The postnatal time points selected for analyzing offspring (P14 and P32) correspond to the suckling and postweaning periods in humans, providing a window for modeling how MDCF-2–associated microbiome repair influences gut function and growth.

Our model can be used to extend these analyses in the future. Integrating the results of microbial RNA-seq with nontargeted and targeted mass spectrometry provides an opportunity to i) nominate candidate effectors and biomarkers of tuft cell responses to microbiome repair and ii) further ascertain the contributions of tuft cells to various facets of intestinal responses/adaptations to undernourished states (including gut mucosal barrier function). The efficiency of mother-to-offspring transmission of MAGs whose abundances were significantly associated with ponderal growth (WLZ) in the clinical trial was high, ranging from 86 to 98%. In principle, the experimental approach could be generalized and be either retrospective or prospective. For example, microbiome samples could be obtained from a given trial participant or combined from multiple trial participants with established robust versus weak responses to treatment. The microbiomes could also be from children representing different ages or different time points during and following treatment, or with different types/degrees of comorbidities. Moreover, sampled microbiomes could be utilized to compare responses to additional treatments not incorporated into the original trial design or to compare responses of trial participants to those obtained from other untreated populations.

The current study deliberately deferred testing the combination of unrepaired intact community and MDCF-2, or repaired intact community and pretreatment diets, largely because directly determining the effects of food components and community membership on expressed microbial and host functions requires an ability to deliberately and precisely manipulate these two factors. Given that microbial communities adapt rapidly to dietary changes (47), introducing a “mismatch” between a microbiome’s repair status and diet (i.e., combining an unrepaired microbiome with a MDCF-2-supplemented weaning diet or a repaired microbiome with an unsupplemented weaning diet) could yield “hybrid” communities that do not reflect the states in clinical trial participants that were the focus of our mechanistic analyses.

A previous study from our group that used a simplified microbial community comprised of a consortium of cultured genome-sequenced age- and growth-discriminatory organisms cultured from the study population did not detect the effects of MDCF-2 treatment on tuft and goblet cells (8). However, integrating findings from reverse translation experiments employing intact microbiomes with experiments involving defined consortia should enable further refinement of the composition of defined communities and tests of hypotheses about the mechanisms by which microbiome repair affects microbial community and host biology. For example, adding enteropathogens cultured from trial participants to the defined community of cultured age- and growth-discriminatory bacterial taxa that we previously examined in gnotobiotic mice (8), represents one approach for deciphering the mechanisms by which the MDCF-2 and/or future prebiotic/synbiotic formulations designed to effect microbiome repair and promote growth can result in pathogen exclusion and affect components of the mucosal barrier.

## Methods

### Gnotobiotic Mouse Studies.

Gnotobiotic mouse experiments were conducted using protocols approved by the Washington University Animal Studies Committee. Germ-free C57BL/6J mice were housed in plastic flexible film isolators (Class Biologically Clean Ltd) maintained at 23 °C with a strict 12-h light/dark cycle (lights on at 0600 h). To support natural nesting behaviors and provide enrichment, autoclaved paper “shepherd shacks” were included in each cage. Weaning diets supplemented with Mirpur-18 and MDCF-2 were prepared as described previously (8) and sterilized via gamma irradiation (30 to 50 KGy). The sterility of the pellets was confirmed by culturing them in LYBHI medium and Wilkins-Chalgren Anaerobe Broth under aerobic and anaerobic conditions for 7 d at 37 °C, followed by plating on LYBHI and blood-agar plates. Nutritional analysis was conducted by Nestlé Purina Analytical Laboratories (St. Louis, MO) (Dataset S1). Mice were euthanized in a carbon dioxide chamber (delivered at 30-70% chamber volume displacement per minute), in accordance with the Washington University Animal Euthanasia Policy (https://research.washu.edu/animal-euthanasia-policy/).

### Donor Selection and Preparation of Fecal Microbial Community Samples.

The study used fecal samples collected prior to treatment (day 0) and at the end of treatment (day 90) from two participants (see Dataset S2*C* for donor characteristics) in the MDCF-2 arm of a previously reported randomized controlled clinical trial (ClinicalTrials.gov identifier NCT04015999) conducted in Dhaka, Bangladesh. This trial was approved by the Ethical Review Committee at the icddr,b [Research Protocol # PR-18073 (5)]. Fecal samples were coded and provided with informed consent for future use to Washington University under MTA with a Code Access Agreement (Washington University Human Research Protection Office (HRPO) Study Protocol IRB ID# 201111065). Donors were selected based on the following criteria: i) their WLZ improvement ranked within the upper quartile among all participants in the MDCF-2 arm; ii) they exhibited the greatest number and largest magnitude of increases in bacterial taxa positively associated with WLZ in response to MDCF-2 treatment; and iii) they showed the most extensive reductions in both number and magnitude of taxa negatively associated with WLZ (8). Refer to *SI Appendix* for details regarding preparation of fecal microbial community samples.

### Metagenomic Sequencing.

DNA was isolated from cecal contents and fecal samples by bead beating and phenol-chloroform extraction and further purified using the QIAquick 96 PCR Purification Kit. Shotgun sequencing libraries were prepared using the Nextera XT DNA Library Prep Kit and sequenced using an Illumina NextSeq platform (2 × 150 bp paired-end reads). Sequencing yielded an average of 6.15 × 10^8^ ± 3.3 × 10^5^ reads per sample from the donor 1 experiment and 6.04 × 10^8^ ± 2.24 × 10^5^ reads per sample from the donor 2 experiment (mean ± SD; Dataset S2*A*). Refer to *SI Appendix* for details regarding data analysis.

### Microbial RNA Sequencing (Microbial RNA-Seq).

RNA was isolated from cecal contents collected from P32 pups using a phenol-chloroform-based method. cDNA libraries were generated from isolated RNA samples using the “Total RNA Prep with Ribo-Zero Plus” kit (Illumina). Barcoded libraries were sequenced (Illumina NovaSeq instrument; 2 × 150 bp paired-end reads; n = 8 samples for the two arms of experiments involving a given donor; 5.5 × 10^7^ ± 2.0 × 10^6^ raw reads/sample (mean ± SD) in the donor 1 experiment and 5.4 × 10^7^ ± 4.7 × 10^6^ raw reads/sample in donor 2 experiment; Dataset S3*A*]. Refer to *SI Appendix* for details regarding data analysis.

### Single-Nucleus RNA-Sequencing (snRNA-seq) and Analysis.

The small intestine was divided into thirds; 4-cm-long segments were recovered from each third and defined as duodenum, jejunum, and ileum. Epithelial nuclei were isolated by adopting a previously described method (48). 10,000 nuclei per sample were subjected to gel bead-in-emulsion (GEM) generation, reverse transcription, and library construction according to the protocol provided in the 3’ gene expression v3.1 kit manual (10× Genomics). Balanced libraries were sequenced [Illumina NovaSeq S4; 2 × 150bp paired-end reads; 6.5 ± 1.6 × 10^4^ reads/nucleus (mean ± SD); Dataset S4*A*]. Detailed methods for nuclei isolation and data analysis are described in *SI Appendix*.

### Histology and Immunostaining.

Duodenal, jejunal, and ileal tissues, collected from P32 pups in both donor 1 and donor 2 experiments, were fixed in 10% formalin, embedded in paraffin, and sectioned into 5 μm-thick slices along the longitudinal axis of the small intestine. Refer to *SI Appendix* for immunostaining, image acquisition, and analyses.

### Mass Spectrometry.

Short-chain fatty acids and eicosanoids were quantified by gas chromatography-mass spectrometry (GC-MS) and High Performance Liquid Chromatography Triple Quadrupole (QqQ) mass spectrometry, respectively. Refer to *SI Appendix* for details.

## Supplementary Material

Appendix 01 (PDF)

Dataset S01 (XLSX)

Dataset S02 (XLSX)

Dataset S03 (XLSX)

Dataset S04 (XLSX)

Dataset S05 (XLSX)

Dataset S06 (XLSX)

Dataset S07 (XLSX)

## Data Availability

Shotgun sequencing, microbial RNA-Seq, and snRNA-Seq datasets have been deposited in the NIH Sequence Read Archive (Accession #: PRJNA1262995) (49). Fecal specimens used in these studies were provided to Washington University under a materials transfer agreement with icddr,b.

## References

[1] S. Subramanian , Persistent gut microbiota immaturity in malnourished Bangladeshi children. Nature **510**, 417–421 (2014). 24896187 10.1038/nature13421 PMC4189846

[2] M. Selimoğlu , Nutritional support in malnourished children with compromised gastrointestinal function: Utility of peptide-based enteral therapy. Front. Pediatr. **9**, 610275 (2021). 34164352 10.3389/fped.2021.610275 PMC8215107

[3] A. M. Mowat, W. W. Agace, Regional specialization within the intestinal immune system. Nat. Rev. Immunol. **14**, 667–685 (2014). 25234148 10.1038/nri3738

[4] J. L. Gehrig , Effects of microbiota-directed foods in gnotobiotic animals and undernourished children. Science **365**, eaau4732 (2019). 31296738 10.1126/science.aau4732 PMC6683325

[5] R. Y. Chen , A microbiota-directed food intervention for undernourished children. N. Engl. J. Med. **384**, 1517–1528 (2021). 33826814 10.1056/NEJMoa2023294 PMC7993600

[6] M. C. Hibberd , Bioactive glycans in a microbiome-directed food for children with malnutrition. Nature **625**, 157–165 (2024). 38093016 10.1038/s41586-023-06838-3 PMC10764277

[7] S. J. Hartman , A microbiome-directed therapeutic food for children recovering from severe acute malnutrition. Sci. Transl. Med. **16**, eadn2366 (2024). 39356745 10.1126/scitranslmed.adn2366 PMC11572952

[8] H.-W. Chang , *Prevotella copri* and microbiota members mediate the beneficial effects of a therapeutic food for malnutrition. Nat. Microbiol. **9**, 922–937 (2024). 38503977 10.1038/s41564-024-01628-7 PMC10994852

[9] A. Wagner , Metabolic modeling of single Th17 cells reveals regulators of autoimmunity. Cell **184**, 4168–4185.e21 (2021). 34216539 10.1016/j.cell.2021.05.045 PMC8621950

[10] I. Thiele , A community-driven global reconstruction of human metabolism. Nat. Biotechnol. **31**, 419–425 (2013). 23455439 10.1038/nbt.2488 PMC3856361

[11] L. Chen , HNF4 regulates fatty acid oxidation and is required for renewal of intestinal stem cells in mice. Gastroenterology **158**, 985–999.e9 (2020). 31759926 10.1053/j.gastro.2019.11.031 PMC7062567

[12] R. R. Stine , PRDM16 maintains homeostasis of the intestinal epithelium by controlling region-specific metabolism. Cell Stem Cell **25**, 830–845.e8 (2019). 31564549 10.1016/j.stem.2019.08.017 PMC6898778

[13] F. Blachier, C. Boutry, C. Bos, D. Tomé, Metabolism and functions of l-glutamate in the epithelial cells of the small and large intestines12. Am. J. Clin. Nutr. **90**, 814S–821S (2009). 19571215 10.3945/ajcn.2009.27462S

[14] C. Schneider, C. E. O’Leary, R. M. Locksley, Regulation of immune responses by tuft cells. Nat. Rev. Immunol. **19**, 584–593 (2019). 31114038 10.1038/s41577-019-0176-x PMC8331098

[15] D. Ambort , Calcium and pH-dependent packing and release of the gel-forming MUC2 mucin. Proc. Natl. Acad. Sci. U.S.A. **109**, 5645–5650 (2012). 22451922 10.1073/pnas.1120269109 PMC3326483

[16] J. B. Silverman, P. N. Vega, M. J. Tyska, K. S. Lau, Intestinal tuft cells: Morphology, function, and implications for human health. Annu. Rev. Physiol. **86**, 479–504 (2024). 37863104 10.1146/annurev-physiol-042022-030310 PMC11193883

[17] F. Gerbe , Intestinal epithelial tuft cells initiate type 2 mucosal immunity to helminth parasites. Nature **529**, 226–230 (2016). 26762460 10.1038/nature16527 PMC7614903

[18] M. R. Howitt , Tuft cells, taste-chemosensory cells, orchestrate parasite type 2 immunity in the gut. Science **351**, 1329–1333 (2016). 26847546 10.1126/science.aaf1648 PMC5528851

[19] J. von Moltke, M. Ji, H.-E. Liang, R. M. Locksley, Tuft-cell-derived IL-25 regulates an intestinal ILC2-epithelial response circuit. Nature **529**, 221–225 (2016). 26675736 10.1038/nature16161 PMC4830391

[20] A. A. Arzamasov , Integrative genomic reconstruction reveals heterogeneity in carbohydrate utilization across human gut bifidobacteria. Nat. Microbiol. **10**, 2031–2047 (2025). 40670725 10.1038/s41564-025-02056-x PMC12313528

[21] M. Fallani , Determinants of the human infant intestinal microbiota after the introduction of first complementary foods in infant samples from five European centres. Microbiology **157**, 1385–1392 (2011). 21330436 10.1099/mic.0.042143-0

[22] T. Odamaki , Age-related changes in gut microbiota composition from newborn to centenarian: A cross-sectional study. BMC Microbiol. **16**, 90 (2016). 27220822 10.1186/s12866-016-0708-5 PMC4879732

[23] M. Kasendra , Development of a primary human small intestine-on-a-chip using biopsy-derived organoids. Sci. Rep. **8**, 2871 (2018). 29440725 10.1038/s41598-018-21201-7 PMC5811607

[24] A. S. Raman , A sparse covarying unit that describes healthy and impaired human gut microbiota development. Science **365**, eaau4735 (2019). 31296739 10.1126/science.aau4735 PMC6683326

[25] D. R. Donohoe , The microbiome and butyrate regulate energy metabolism and autophagy in the mammalian colon. Cell Metab. **13**, 517–526 (2011). 21531334 10.1016/j.cmet.2011.02.018 PMC3099420

[26] E. C. Aguilar , Butyrate impairs atherogenesis by reducing plaque inflammation and vulnerability and decreasing NFκB activation. Nutr. Metab. Cardiovasc. Dis. **24**, 606–613 (2014). 24602606 10.1016/j.numecd.2014.01.002

[27] E. M. Eshleman , Microbiota-derived butyrate restricts tuft cell differentiation via histone deacetylase 3 to modulate intestinal type 2 immunity. Immunity **57**, 319–332 (2024). 38295798 10.1016/j.immuni.2024.01.002 PMC10901458

[28] K. Matter, S. Aijaz, A. Tsapara, M. S. Balda, Mammalian tight junctions in the regulation of epithelial differentiation and proliferation. Curr. Opin. Cell Biol. **17**, 453–458 (2005). 16098725 10.1016/j.ceb.2005.08.003

[29] Y. Maruyama , Metabolic fate of unsaturated glucuronic/iduronic acids from glycosaminoglycans. J. Biol. Chem. **290**, 6281–6292 (2015). 25605731 10.1074/jbc.M114.604546 PMC4358265

[30] F. O’Leary, S. Samman, Vitamin B12 in health and disease. Nutrients **2**, 299–316 (2010). 22254022 10.3390/nu2030299 PMC3257642

[31] D. R. Herbert , Intestinal epithelial cell secretion of RELM-β protects against gastrointestinal worm infection. J. Exp. Med. **206**, 2947–2957 (2009). 19995957 10.1084/jem.20091268 PMC2806463

[32] Z. Xiong , Intestinal tuft-2 cells exert antimicrobial immunity via sensing bacterial metabolite N-undecanoylglycine. Immunity **55**, 686–700.e7 (2022). 35320705 10.1016/j.immuni.2022.03.001

[33] S. K. Linden, P. Sutton, N. G. Karlsson, V. Korolik, M. A. McGuckin, Mucins in the mucosal barrier to infection. Mucosal Immunol. **1**, 183–197 (2008). 19079178 10.1038/mi.2008.5 PMC7100821

[34] L. E. Tailford, E. H. Crost, D. Kavanaugh, N. Juge, Mucin glycan foraging in the human gut microbiome. Front. Genet. **6**, 81 (2015). 25852737 10.3389/fgene.2015.00081 PMC4365749

[35] B. Dolan, A. Ermund, B. Martinez-Abad, M. E. V. Johansson, G. C. Hansson, Clearance of small intestinal crypts involves goblet cell mucus secretion by intracellular granule rupture and enterocyte ion transport. Sci. Signal. **15**, eabl5848 (2022). 36126118 10.1126/scisignal.abl5848 PMC9749883

[36] C. Aurrecoechea , EuPathDB: A portal to eukaryotic pathogen databases. Nucleic Acids Res. **38**, D415–D419 (2010). 19914931 10.1093/nar/gkp941 PMC2808945

[37] B. Buchfink, C. Xie, D. H. Huson, Fast and sensitive protein alignment using DIAMOND. Nat. Methods **12**, 59–60 (2015). 25402007 10.1038/nmeth.3176

[38] R. Browaeys , MultiNicheNet: A flexible framework for differential cell-cell communication analysis from multi-sample multi-condition single-cell transcriptomics data. bioRxiv [Preprint] (2023), 10.1101/2023.06.13.544751 (Accessed 14 June 2023).

[39] I. Mostafa , A microbiota-directed complementary food intervention in 12–18-month-old Bangladeshi children improves linear growth. Ebiomedicine **104**, 105166 (2024). 38833839 10.1016/j.ebiom.2024.105166 PMC11179573

[40] J. von Moltke , Leukotrienes provide an NFAT-dependent signal that synergizes with IL-33 to activate ILC2s. J. Exp. Med. **214**, 27–37 (2016). 28011865 10.1084/jem.20161274 PMC5206504

[41] J. W. McGinty , Tuft-cell-derived leukotrienes drive rapid anti-helminth immunity in the small intestine but are dispensable for anti-protist immunity. Immunity **52**, 528–541.e7 (2020). 32160525 10.1016/j.immuni.2020.02.005 PMC7469474

[42] M. Keshavarz , Cysteinyl leukotrienes and acetylcholine are biliary tuft cell cotransmitters. Sci. Immunol. **7**, eabf6734 (2022). 35245090 10.1126/sciimmunol.abf6734

[43] K. E. DelGiorno , Tuft cells inhibit pancreatic tumorigenesis in mice by producing prostaglandin D2. Gastroenterology **159**, 1866–1881.e8 (2020). 32717220 10.1053/j.gastro.2020.07.037 PMC7680354

[44] P. V. Beum, H. Basma, D. R. Bastola, P.-W. Cheng, Mucin biosynthesis: Upregulation of core 2 β1,6 N -acetylglucosaminyltransferase by retinoic acid and Th2 cytokines in a human airway epithelial cell line. Am. J. Physiol. Lung Cell **288**, L116–L124 (2005). 10.1152/ajplung.00370.2003 15591039

[45] K. Niimi, E. Takahashi, Reduced differentiation of intestinal epithelial cells in wasting marmoset syndrome. J. Vet. Med. Sci. **83**, 784–792 (2021). 33731497 10.1292/jvms.20-0532 PMC8182325

[46] C. A. Cowardin , Mechanisms by which sialylated milk oligosaccharides impact bone biology in a gnotobiotic mouse model of infant undernutrition. Proc. Natl. Acad. Sci. **116**, 11988–11996 (2019). 31138692 10.1073/pnas.1821770116 PMC6575181

[47] M. S. Kennedy , Diet outperforms microbial transplant to drive microbiome recovery in mice. Nature **642**, 747–755 (2025). 40307551 10.1038/s41586-025-08937-9

[48] J. T. Gaublomme , Nuclei multiplexing with barcoded antibodies for single-nucleus genomics. Nat. Commun. **10**, 2907 (2019). 31266958 10.1038/s41467-019-10756-2 PMC6606589

[49] Y. Wang, H.-W. Chang, J. I. Gordon, Deciphering effects of gut microbiome repair achieved in undernourished Bangladeshi children in gnotobiotic mice. SRA. https://www.ncbi.nlm.nih.gov/bioproject/PRJNA1262995. Deposited 14 May 2025.

