# Supplementary material for: Using gnotobiotic mice to decipher effects of gut microbiome repair in undernourished children on tuft and goblet cell function
Source: Proc Natl Acad Sci U S A. 2025 Nov 25;122(48):e2523178122. doi: 10.1073/pnas.2523178122 (PMC12685025; doi:10.1073/pnas.2523178122)
Supplement: Supplementary file 1 — Appendix 01 (PDF) [file pnas.2523178122.sapp.pdf]

PNAS

Supporting Information for

Using gnotobiotic mice to decipher effects of gut microbiome repair in undernourished children on tuft and goblet cell function

Yi Wang[1,2,3]*, Hao-Wei Chang[1,2]*, Jiye Cheng[1,2,3], Daniel M. Webber[1,2,3], Hannah M. Lynn[1,2], Matthew C. Hibberd[1,2,3], Clara Kao[1,2], Ishita Mostafa[4], Tahmeed Ahmed[4], Michael J. Barratt[1,2,3], and Jeffrey I. Gordon[1,2,3#]

[1]Edison Family Center for Genome Sciences and Systems Biology, Washington University School of Medicine, St. Louis, MO 63110 USA

[2]Newman Center for Gut Microbiome and Nutrition Research, Washington University School of Medicine, St. Louis, MO 63110 USA

[3]Department of Pathology and Immunology, Washington University School of Medicine, St. Louis, MO 63110 USA

[4]International Centre for Diarrhoeal Disease Research, Bangladesh (icddr,b), Dhaka 1212, Bangladesh

[#]Corresponding author: Jeffrey I. Gordon
Email: jgordon@wustl.edu

This PDF file includes:

Figures S1 to S4
Legends for Datasets S1 to S7
Supplementary Methods
SI References

Other supporting materials for this manuscript include the following:

Datasets S1 to S7

Supplementary Figures

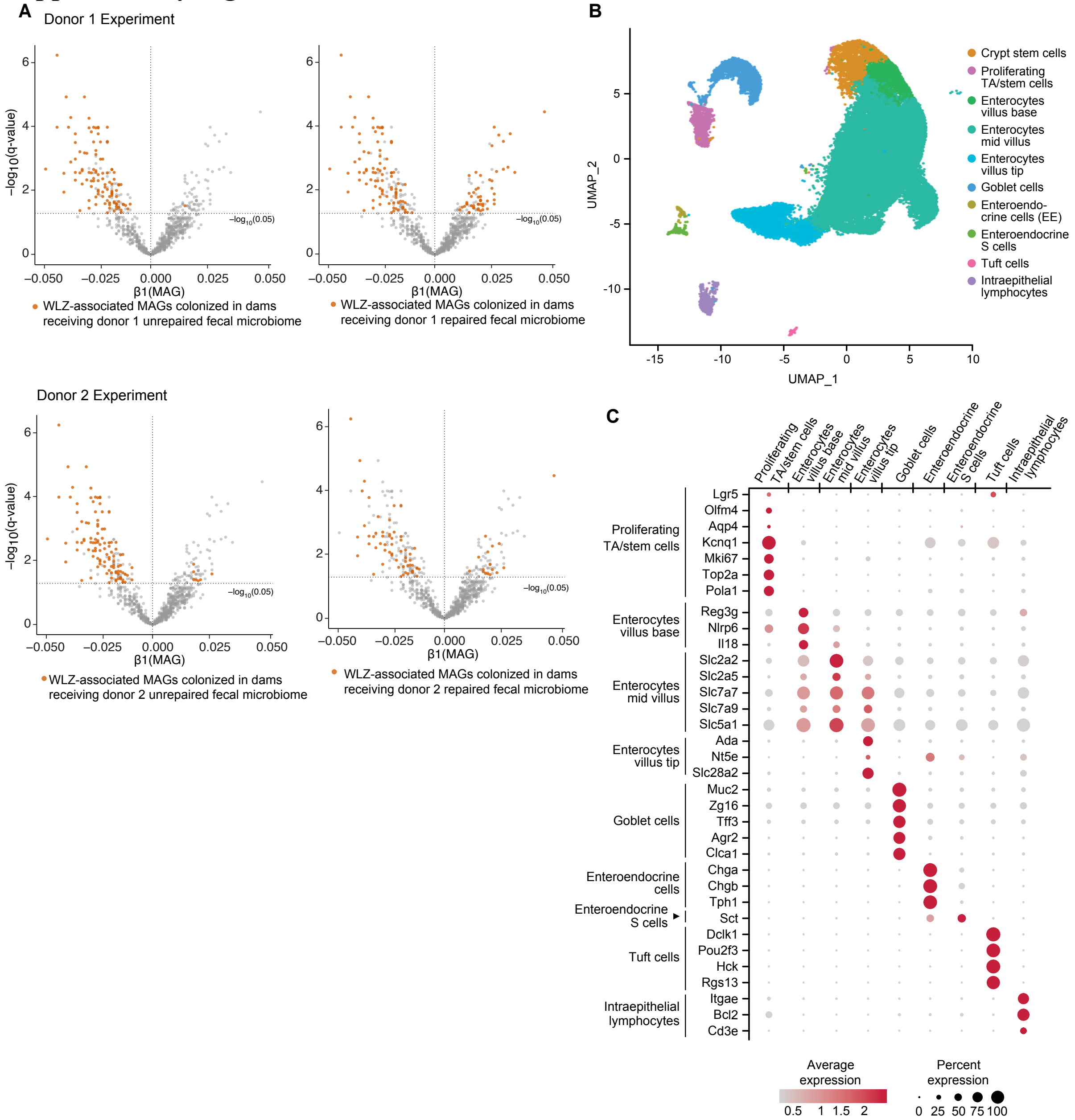

SI Appendix Fig. S1. Volcano plots for WLZ-associated MAGs colonized in dams and snRNA-seq analysis of the small intestinal tissues. A. Volcano plots showing the relationship between WLZ-association effect size (β1 coefficient in the linear model shown in Fig. 1) and statistical significance (–$\log_{10}$ q-value) for all MAGs identified in the clinical trial (gray points). WLZ-associated MAGs that colonized dams are highlighted in orange for each condition: donor 1's unrepaired and repaired microbiome (top left and right subpanels, respectively); donor 2's unrepaired and repaired microbiome (bottom left and right subpanels). **B.** UMAP plot of snRNA-seq dataset generated from jejunal tissues in the donor 1 experiment, showing identified epithelial clusters. **C.** Dot plot illustrating the expression of known marker genes across cell types identified in the UMAP plot in panel A. The color intensity represents the expression level, while the dot size corresponds to the percentage of cells expressing the marker.

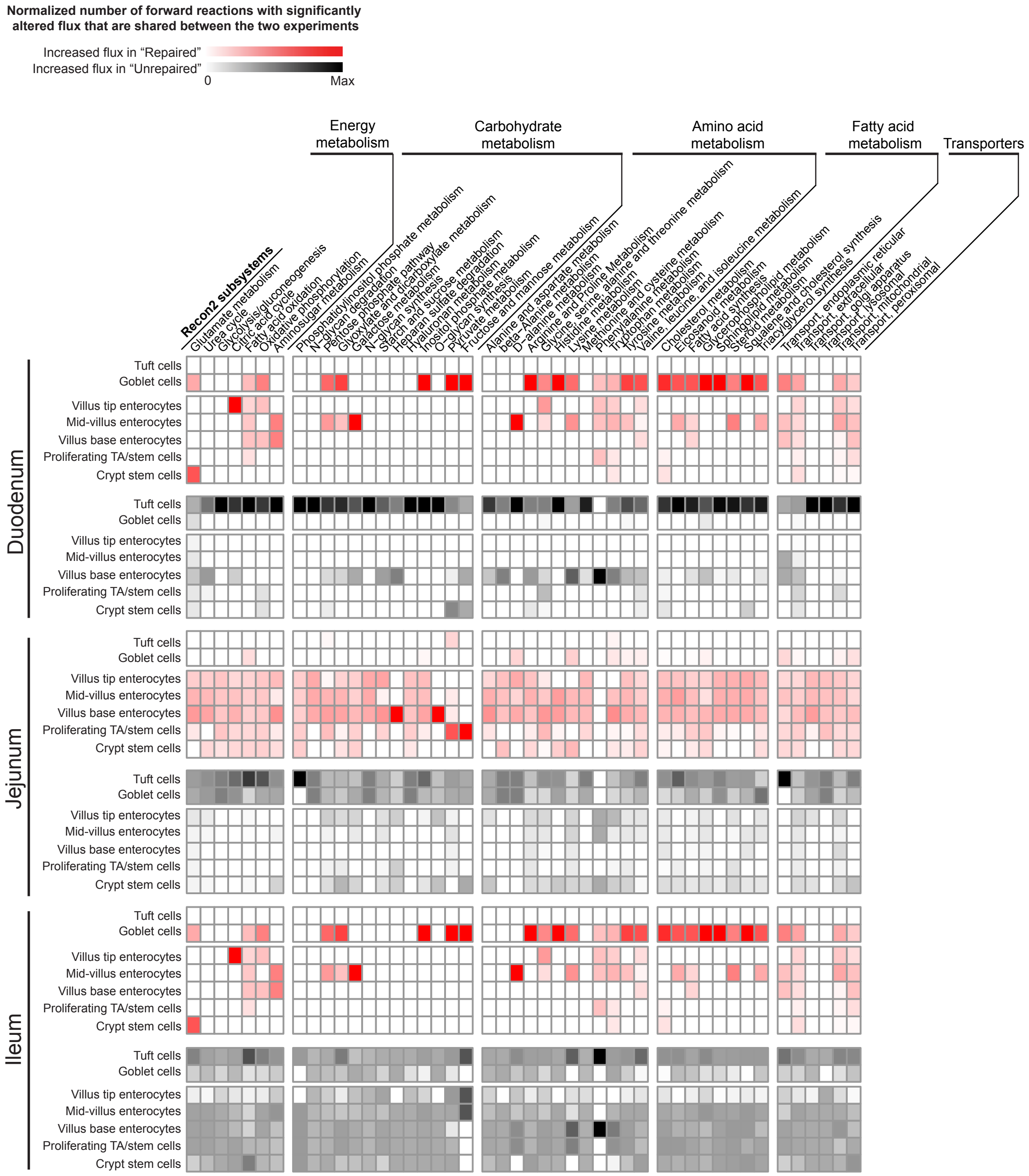

Normalized number of forward reactions with significantly altered flux that are shared between the two experiments
Increased flux in "Repaired"
Increased flux in "Unrepaired"
0
Max
Energy metabolism
Carbohydrate metabolism
Amino acid metabolism
Fatty acid metabolism
Transporters
Recon2 subsystems
Glutamate metabolism
Urea cycle
Glycolysis/gluconeogenesis
Citric acid cycle
Fatty acid oxidation
Oxidative phosphorylation
Aminosugar metabolism
Phosphatidylinositol phosphate metabolism
N-glycan degradation
Pentose phosphate pathway
Glyoxylate and dicarboxylate metabolism
Galactose metabolism
N-glycan synthesis
Starch and sucrose metabolism
Heparan sulfate degradation
Hyaluronan metabolism
Inositol phosphate metabolism
O-glycan synthesis
Pyruvate metabolism
Fructose and mannose metabolism
Alanine and aspartate metabolism
beta-Alanine metabolism
D-alanine metabolism
Arginine and Proline Metabolism
Glycine, serine, alanine and threonine metabolism
Histidine metabolism
Lysine metabolism
Methionine and cysteine metabolism
Phenylalanine metabolism
Tryptophan metabolism
Tyrosine metabolism
Valine, leucine, and isoleucine metabolism
Cholesterol metabolism
Eicosanoid metabolism
Fatty acid synthesis
Glycerophospholipid metabolism
Sphingolipid metabolism
Steroid metabolism
Squalene and cholesterol synthesis
Triacylglycerol synthesis
Transport, endoplasmic reticular
Transport, extracellular
Transport, golgi apparatus
Transport, lysosomal
Transport, mitochondrial
Transport, peroxisomal
Duodenum
Tuft cells
Goblet cells
Villus tip enterocytes
Mid-villus enterocytes
Villus base enterocytes
Proliferating TA/stem cells
Crypt stem cells
Tuft cells
Goblet cells
Villus tip enterocytes
Mid-villus enterocytes
Villus base enterocytes
Proliferating TA/stem cells
Crypt stem cells
Jejunum
Tuft cells
Goblet cells
Villus tip enterocytes
Mid-villus enterocytes
Villus base enterocytes
Proliferating TA/stem cells
Crypt stem cells
Tuft cells
Goblet cells
Villus tip enterocytes
Mid-villus enterocytes
Villus base enterocytes
Proliferating TA/stem cells
Crypt stem cells
Ileum
Tuft cells
Goblet cells
Villus tip enterocytes
Mid-villus enterocytes
Villus base enterocytes
Proliferating TA/stem cells
Crypt stem cells
Tuft cells
Goblet cells
Villus tip enterocytes
Mid-villus enterocytes
Villus base enterocytes
Proliferating TA/stem cells
Crypt stem cells

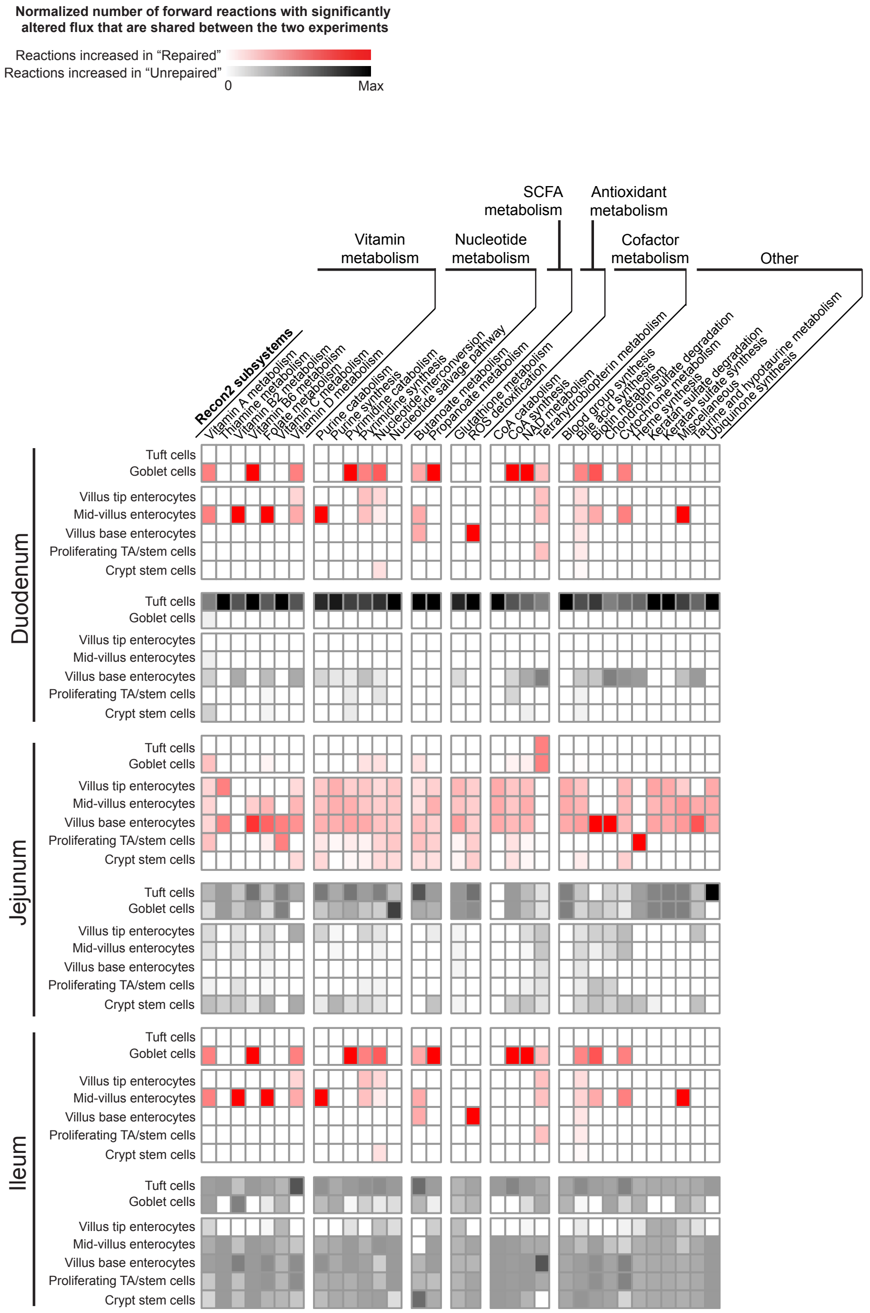

SI Appendix Fig. S2. Normalized number of Compass-predicted metabolic reactions with significantly altered flux in 79 Recon2 subsystems. A. Heatmap representing the normalized number of Compass-predicted forward metabolic reactions with significantly altered flux that are shared between donor 1 and donor 2 experiments. Data are presented for 79 Recon2 metabolic subsystems across all epithelial cell clusters from duodenum, jejunum and ileum. Red indicates the normalized number of reactions with increased flux in the repaired arm, while black indicates the normalized number of reactions with increased flux in the unrepaired arm. Color density represents the number of reactions with significantly increased flux in a given Recon2 subsystem within a specific cell type of an experimental arm, normalized to the total number of such reactions across all epithelial cell clusters in the same experimental arm.

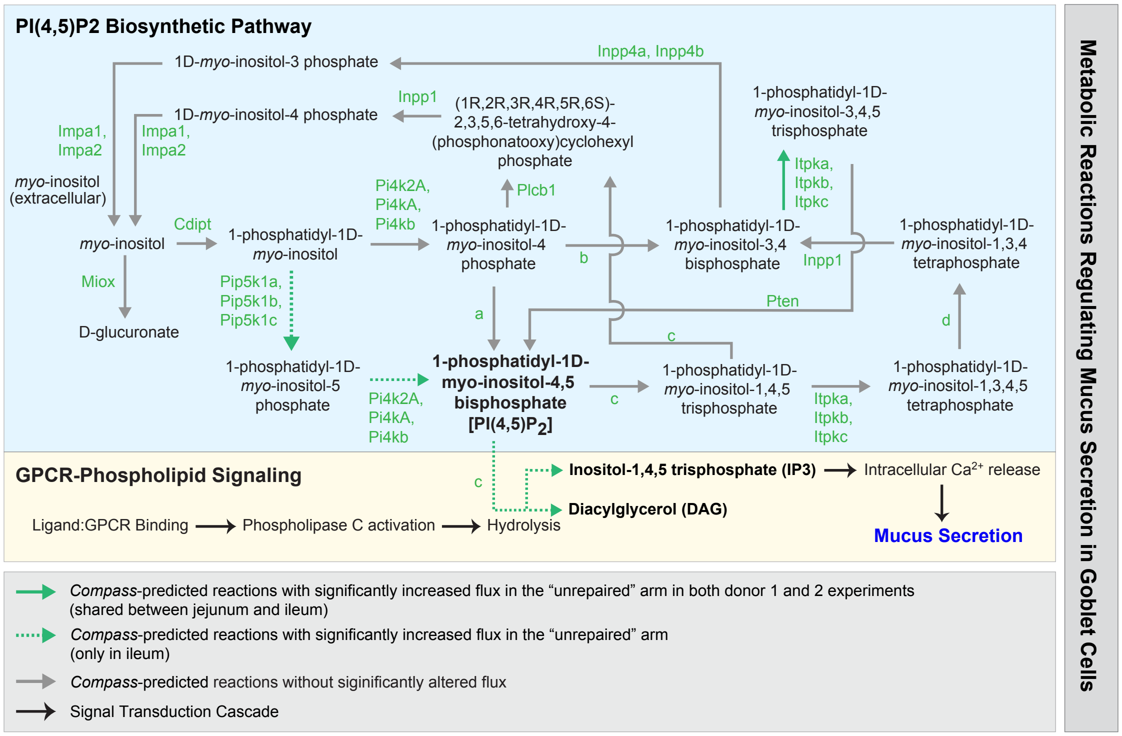

SI Appendix Fig. S3. Metabolic reactions related to glycosylation and secretion of mucin in goblet cells. Schematic representation of reactions involved in PIP2 biosynthesis and GPCR-phospholipid signaling pathways. Green arrows represent reactions with significantly increased flux predicted by *Compass* in both jejunal and ileal goblet cells of the "unrepaired" arm across donor 1 and donor 2 experiments. Green dotted arrows indicate reactions with significantly increased flux in jejunal goblet cells only. Grey arrows denote reactions with no predicted significant changes in flux. Black arrows highlight the signal transduction cascade associated with GPCR-phospholipid signaling.

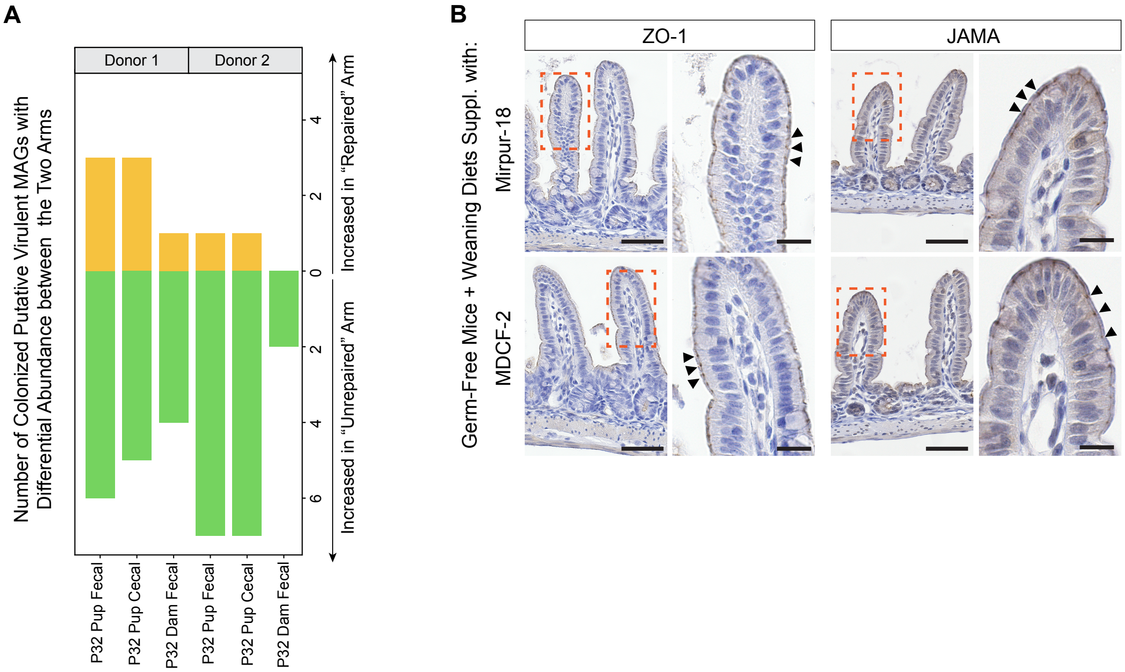

SI Appendix Fig. S4. Analysis of putative virulent MAG and immunocytochemistry of epithelial junction proteins in control germ-free mice. A. Bar blot demonstrating the number of colonized putative virulent MAGs with differential abundance between unrepaired and repaired arms in the fecal and cecal samples of P32 pups in donor 1 and donor 2 experiments. **B.** Representative images showing the distribution of tight junction components ZO-1 and JAMA in the jejunums of P32 germ-free pups fed with weaning diets supplemented with Mirpur-18 and MDCF-2. Brown staining indicates a positive immunoreactive signal, while blue staining represents nuclei counterstained with hematoxylin. The area denoted with the dashed red box is shown at higher power in the adjacent image. Scale bars: 50 μm in the left panels and 20 μm the right panels.

Supplementary Dataset Legends

Dataset S1. Diets used in gnotobiotic mouse studies. (A) Ingredients in each diet module. (B) Representation of modules in the weaning diet supplemented with Mirpur-18 or MDCF-2. (C) Nutritional analysis of the diets.

Dataset S2. Fecal and cecal DNA shotgun sequencing datasets generated from gnotobiotic mouse experiments involving intact uncultured microbiomes from two participants in the proof-of-concept MDCF-2 clinical trial. (A) Sample metadata. (B) Normalized count table of WLZ-associated MAGs in fecal and cecal samples. (C) Maternal transmission of WLZ-associated MAGs. (D) Normalized count table of colonized WLZ-associated MAGs as defined by prevalence > 0.4 and TPM > 20 in cecal samples obtained from P32 pups. (E) DIAMOND alignment results of virulence factors from the Virulence Factor Database (VFDB).

Dataset S3. snRNA-Seq dataset generated from the jejunums of gnotobiotic mice colonized with fecal microbiome samples obtained from two donors in the MDCF-2 clinical trial. (A) Sample metadata. (B) Differentially expressed genes in jejunums (q-value < 0.1; fold-difference > 1.5). (C) Percentage of shared statistically significant `Reaction Direction Combinations` identified by Compass analysis of each cell type in each small intestinal segment between the two donor experiments. (D) Percentage of shared statistically significant `Reaction Direction Combinations` identified by Compass for each cell type in each small intestinal segment between this study and supplementary ref. 9. (E) Epithelial tight junction interactions in the top 10% prioritization score identified by MultiNicheNet analysis.

Dataset S4. Tuft cell density.

Dataset S5. Cecal microbial RNA-seq datasets generated from gnotobiotic mouse experiments involving intact uncultured microbiomes from two participants in the MDCF-2 clinical trial. (A) Sample metadata. (B) mcSEED annotation for transcripts subject to differential expression analysis and GSEA. (C) Shared statistically significantly enriched mcSEED pathways between the two experiments. (D) Leading-edge MAGs that drove the shared enriched mcSEED pathways. (E) Statistically significantly enriched virulence pathways in the two donor experiments. (F) Leading-edge MAGs that drove the enriched virulence pathways in the two donor experiments.

Dataset S6. Targeted mass spectrometric analysis of short-chain fatty acids in the cecal contents of gnotobiotic mice colonized with fecal microbiome samples obtained from two donors in the MDCF-2 clinical trial.

Dataset S7. Goblet cell density.

Supplementary Methods

Preparation of fecal microbial community samples

To prepare intact, uncultured fecal microbial communities, fecal samples were pulverized in liquid nitrogen. Aliquots of pulverized material (300 mg) were transferred to a Coy Chamber under anaerobic conditions (75% N_2, 20% CO_2 and 5% H_2) and resuspended in 4 mL of phosphate buffered saline (PBS) containing 30% glycerol and 0.05% cysteine-HCl. The mixture was vortexed for 2 minutes, pausing for 5 seconds every 30 seconds, and passed through a 100 µm pore-diameter strainer (Corning, catalog number 431752). One milliliter aliquots of the resulting filtrate were placed into crimp-top tubes (Wheaton, catalog number 225175) which were transferred out of the Coy Chamber and stored at -80 ºC until use. A 200 µL aliquot was administered to dams via a 22-gauge oral gavage needle (Cadence Science, catalog number 7901).

Metagenomic sequencing data analysis

Reads were demultiplexed using bcl2fastq (v2.2.0), trimmed with Trim Galore (v0.6.4), and filtered to exclude host-derived sequences using bowtie2 (v2.3.4.1). The remaining reads were aligned using Kallisto (v0.43.0) to the 1000 MAGs that had previously been assembled from participants in the randomized controlled clinical trial (1) to generate a transcript-per-million (TPM) count matrix. Differential abundance analysis between treatment conditions was conducted using DESeq2 (2). MAGs were considered to have colonized the mouse gut if their TPM exceeded 20 and their prevalence across samples was greater than 40%.

To identify putative virulent MAGs, we employed DIAMOND (v2.1.4) (3) to align protein sequences of virulence factors from the Virulence Factor Database (VFDB) (4) against the protein-coding sequences of MAGs identified in P32 dams and pups from "unrepaired" and "repaired" cecal and fecal samples. MAGs were included in the analysis if they met the colonization criteria of TPM >20 and prevalence greater than 40%. A MAG was classified as putatively virulent if it encoded both exotoxins and effector delivery systems, two essential components of bacterial pathogenesis. Alignments were filtered using a bitscore threshold of >200 and an E-value cutoff of $<10^{-100}$.

Reads from shotgun sequencing of cecal DNA samples from P32 pups were filtered to remove host and MAG sequences. We then used Kraken2 (5) to align to EuPathDB of 388 eukaryotic pathogens (release 46; https://veupathdb.org) (6).

Microbial RNA-seq data analysis

Raw reads were processed for quality control and trimming by fastp v0.20.0. Trimmed reads were aligned to mouse mm10 genome using bowtie2 v2.4.2 to filter out host reads. Host-filtered reads were mapped to the 1000 MAGs identified in the clinical study (1) with Kallisto v0.48.0. The resulting Kallisto pseudocount dataset was imported into R v4.1.4.

GSEA was conducted for (i) virulence factor categories from the Virulence Factor Database (VFDB) and (ii) metabolic pathways annotated in the mcSEED database (7), using the fgsea package v1.16.0. A ranked gene list was created by ordering genes included in the differential expression analysis in decreasing order based on $-\log_{10}(P\text{-value})$*sign of $\log_2$(fold-difference). Gene sets were derived from VFDB categories and from mcSEED module 3. Enrichment analysis was performed with 100,000 permutations. Gene sets with an adjusted P-value of <0.1 were considered significantly enriched.

Epithelial nuclei isolation and snRNA-seq data analysis

Each segment from each mouse was opened longitudinally and incubated in ice-cold PBS containing 10 mM EDTA for 10 minutes. The epithelial layer was lifted by gently brushing and scraping with forceps, collected by centrifugation (50 x g for 3 minutes at room temperature) and snap frozen in liquid nitrogen. Nuclei were isolated from the epithelial layer using a method originally described for brain (8) except that the concentration of bovine serum albumin was increased to 2% and RNase inhibitors (Sigma, catalog number 3335402001) to 0.2U/µL in both the lysis and resuspension buffers.

Read alignment, feature-barcode matrices and quality controls were processed using the CellRanger 5.0 pipeline with the '--include-introns' option to ensure intronic reads would map to the mouse reference genome (GRCm38/mm10). Nuclei with over 2.5% reads from mitochondria-encoded genes or ribosomal protein genes were excluded from downstream analysis. Sample integration, count normalization, cell clustering and marker gene identification was performed using Seurat (v4.0). Filtered feature-barcode matrices were converted into Seurat objects using the CreateSeuratObject function (minimum criteria: 5 cells and 200 features). Sample normalization and integration were performed with Seurat (v4.0). The integrated dataset was subjected to unsupervised clustering using the shared nearest-neighbor graph-based algorithms (FindNeighbors and FindClusters; dimensions = 1:30, resolution = 0.8). Cell clusters were manually annotated based on the expression of known marker genes. Differential gene expression across experimental conditions was analyzed based on the "pseudobulk" approach as described previously (9).

MultiNicheNet v2.0.0 (10) was applied to snRNA-seq datasets generated from the duodenum, jejunum, and ileum in both donor 1 and donor 2 experiments. MultiNicheNet enables inference of condition-specific ligand-receptor interaction changes across defined sender and receiver cell populations. For every ligand-receptor pair within a sender–receiver cell type combination, a prioritization score is computed based on (i) $\log_2$-fold change of the ligand expression in sender cells, (ii) $\log_2$-fold change of the receptor expression in the receiver cells, (iii) $-\log_{10}$ *P*-value times the sign of the fold change in the ligand expression in sender cells, (iv) $-\log_{10}$ *P*-value times the sign of the fold change in the receptor expression in receiver cells, (v) ligand activity score based on the differential expression of the target genes in the receiver cells, (vi) mean expression of the ligand in sender cells, (vii) mean expression of the receptor in receiver cells, and (viii) the proportion of samples in which both ligand and receptor are expressed in 10% of sender and receiver cells. The higher the prioritization score, the more confidently the model predicts that this ligand-receptor pair is altered between experimental conditions.

snRNA-Seq data from seven epithelial cell clusters (crypt stem cells, proliferating transit amplifying cells, villus-base enterocytes, mid-villus enterocytes, villus tip enterocytes, tuft cells, and goblet cells) were subjected to Compass-based *in silico* metabolic flux analysis (version 0.9.10). Compass assigned reaction scores to each Recon 2 reaction for each cell cluster. Reaction scores were filtered based on the criteria described in our previous publication (9). Briefly, only Recon 2 reactions that are supported by biochemical evidence (defined by Recon2 as having a confidence level of 4) and have complete enzymatic information for the reaction were advanced to the follow-on analysis. This filtering yielded 2,075 pass filter reactions in 83 Recon 2 subsystems. For each Recon 2 reaction, a Mann-Whitney U test was used to test the statistical significance of the difference in flux between the unrepaired and repaired arm. *P* values from Mann-Whitney U tests were adjusted for multiple comparisons with the Benjamini–Hochberg method. For reversible reactions, a treatment condition may lead to statistically significant increases or decreases in both the forward and reverse directions within a specific cell cluster. To determine the net directionality in such cases, we compared reaction scores between the two directions. If the score of the forward reaction exceeded that of the reverse reaction, the reaction was classified as net forward; if the converse was true, it was classified as net reverse.

Histology and immunostaining

Immunostaining was performed using primary antibodies direct against DCLK1 (Cell Signaling Technology, catalog number 62257S, 1:300 dilution in PBS/0.1% Triton-X), ZO-1 (Abcam, catalog number ab221546, 1:200 dilution), JAMA (ThermoFisher, catalog number 36-1700, 1:400 dilution) and MUC2 (Thermo Fisher, catalog number PA5-21329, 1:500 dilution), followed by species-appropriate fluorophore-conjugated or HRP-conjugated secondary antibodies and DAB-based color development.

Bright field images of whole slides were acquired using a Hamamatsu NanoZoomer 2.0-HT system. Fluorescent whole-slide images were obtained by a 3DHISTECH Panoramic P250 Flash III slide scanner. Tuft cell density in a given small intestinal segment (duodenum, jejunum or ileal) was determined by using a pixel classifier of QuPath v0.5.1 (11). For each intestinal segment from a given animal, we selected the three best preserved and oriented regions, each containing a minimum of 20

crypt–villus units (‘regions of interest’, ROI). The longitudinal length of each tissue segment in the ROI was determined by drawing a smooth line along the muscularis propria. Tuft cell density was calculated by dividing the total number of tuft cells across the three ROIs by the summed tissue length. Goblet cell number (MUC2+) and total cell number (DAPI+) were quantified using the Cellpose Cyto3 model (12), applied to three ROIs per intestinal region selected using the same criteria as for tuft cell analysis. Goblet cell density in a given small intestinal region was calculated as the total number of MUC2+ cells divided by the total number of DAPI+ cells across the three ROIs. All quantification was performed without double blinding.

Mass spectrometry

Short-chain fatty acids were quantified by gas chromatography-mass spectroscopy (GC-MS). Aliquots of flash-frozen cecal contents were weighed and supplemented with 10 µL of a mixture of internal standards (20 mM of acetic acid-$^{13}C_2$,d_4, propionic acid-d_6, butyric acid-$^{13}C_4$, lactic acid-3,3,3- d_3, and succinic acid-$^{13}C_4$). After the addition of 20 µL of 33% HCl and 1 mL diethyl ether, the mixture was vortexed vigorously for 10 minutes and centrifuged at 4,000 × g for 5 minutes. The supernatant was transferred to another vial and a second diethyl ether extraction was performed. After combining the two ether extracts, a 60 µL aliquot was removed, combined with 20 µL N-tert-butyldimethylsilyl-N-methyltrifluoroacetamide (MTBSTFA) in a GC auto-sampler vial with a 200 µL glass insert, and incubated for 2 h at room temperature. Derivatized samples (1 µL) were injected with 15:1 split into an Agilent 7890B/5977B GC-MS system.

For measurement of eicosanoids, frozen intestinal tissue segments were weighed in Matrix D tubes (MP Biomedicals; catalog number 6913050) followed by addition of 19 volumes of ice-cold methanol (microliters per milligram wet weight of tissue). Samples were homogenized (eight rounds each for 1 minute at room temperature using a BioSpec mini-bead beater with cooling on ice for 1 minute between each round). The resulting homogenates were centrifuged at 12,000 × g for 5 minutes at 4 °C. A 300-µL aliquot of each supernatant was transferred to a new tube and dried in a centrifugal evaporator (LabConco CentriVap). Dried samples were resuspended in 100 µL of 50% methanol and centrifuged at 12,000 × g for 1 minute at 4 °C to ensure that no particulate matter is carried forward to the next step. An 80 µL aliquot of each supernatant was carefully transferred without creating bubbles into an Agilent sample vial (with fixed insert) and 5 µL was injected into a 1290 Infinity II UHPLC system coupled to a 6470 Triple Quadrupole (QqQ) mass spectrometer equipped with a Jet Stream electrospray ionization source (Agilent Technologies).

Supplementary References

1. M. C. Hibberd, *et al.*, Bioactive glycans in a microbiome-directed food for children with malnutrition. *Nature* **625**, 157–165 (2024).
2. M. I. Love, W. Huber, S. Anders, Moderated estimation of fold change and dispersion for RNA-seq data with DESeq2. *Genome Biology* **15**, 550 (2014).
3. B. Buchfink, C. Xie, D. H. Huson, Fast and sensitive protein alignment using DIAMOND. *Nat Methods* **12**, 59–60 (2015).
4. B. Liu, D. Zheng, S. Zhou, L. Chen, J. Yang, VFDB 2022: a general classification scheme for bacterial virulence factors. *Nucleic Acids Res* **50**, D912–D917 (2021).
5. D. E. Wood, J. Lu, B. Langmead, Improved metagenomic analysis with Kraken 2. *Genome Biology* **20**, 257 (2019).
6. C. Aurrecoechea, *et al.*, EuPathDB: a portal to eukaryotic pathogen databases. *Nucleic Acids Res* **38**, D415-419 (2010).
7. A. A. Arzamasov, *et al.*, Integrative genomic reconstruction reveals heterogeneity in carbohydrate utilization across human gut bifidobacteria. *Nat Microbiol* **10**, 2031–2047 (2025).
8. J. T. Gaublomme, *et al.*, Nuclei multiplexing with barcoded antibodies for single-nucleus genomics. *Nat Commun* **10**, 2907 (2019).
9. H.-W. Chang, *et al.*, Prevotella copri and microbiota members mediate the beneficial effects of a therapeutic food for malnutrition. *Nat Microbiol* **9**, 922–937 (2024).
10. R. Browaeys, *et al.*, MultiNicheNet: a flexible framework for differential cell-cell communication analysis from multi-sample multi-condition single-cell transcriptomics data. *bioRxiv* (2023). https://doi.org/10.1101/2023.06.13.544751.
11. P. Bankhead, *et al.*, QuPath: Open source software for digital pathology image analysis. *Sci Rep* **7**, 16878 (2017).
12. C. Stringer, M. Pachitariu, Cellpose3: one-click image restoration for improved cellular segmentation. Nat Methods **22**, 592-599 (2025).
